# A new alvarezsaurid dinosaur from the Nemegt Formation of Mongolia

**DOI:** 10.1038/s41598-019-52021-y

**Published:** 2019-10-29

**Authors:** Sungjin Lee, Jin-Young Park, Yuong-Nam Lee, Su-Hwan Kim, Junchang Lü, Rinchen Barsbold, Khishigjav Tsogtbaatar

**Affiliations:** 1 School of Earth and Environmental Sciences, Seoul National University, Seoul, 08826 Republic of Korea; 2 Institute of Geology, Chinese Academy of Geological Sciences, Beijing, 100037 China; 3 Institute of Paleontology and Geology, Mongolian Academy of Sciences, Ulaanbaatar, 15160 Mongolia

**Keywords:** Taxonomy, Palaeontology

## Abstract

Alvarezsaurid diversity has been markedly increased by recent discoveries from China. However, the number of alvarezsaurid specimens in the Nemegt Formation of Mongolia remained low since the initial report on *Mononykus olecranus* in 1993. Here we report three new alvarezsaurid specimens from this formation, which were associated with each other and also with multiple oviraptorid skeletons in a small multi-species assemblage. Two of the alvarezsaurid specimens represent a new taxon, *Nemegtonykus citus* gen. et sp. nov., which is mainly distinguished from other alvarezsaurids by the first sacral vertebra with a subtrapezoidal lamina, the second sacral centrum which is directly co-ossified with ilium, the posterodorsally oriented postacetabular process of ilium, and partial co-ossification between metatarsals II and IV. The other specimen is very similar to *M. olecranus* in morphology and referred to cf. *Mononykus* sp. Our phylogenetic analysis recovered *Nemegtonykus* as a parvicursorine forming a polytomy with several other taxa from the Gobi Desert. The presence of three alvarezsaurid individuals in the same locality indicates that the abundance of alvarezsaurids have been greatly underestimated in the Nemegt dinosaur faunas.

## Introduction

Alvarezsaurs are an extraordinary group of animals which are, in general, characterized by greatly shortened forelimbs with an enlarged medial-most manual digit (particularly parvicursorines) and contrastingly elongate distal hind limbs with a specialized arctometatarsal condition in derived forms^1–14^. Their records span across four continents, but most taxa are from either Asia^1,6,8,9,11,13–22^ or South America^3,12,23–26^, with only fragmentary specimens from North America^5,27^ and Europe^28^. Despite their nearly global distribution, alvarezsaur fossils have been extremely rare compared to other contemporary dinosaurs. The rarity and a lack of complete alvarezsaur specimens have led to many difficulties in interpreting of their ecology or phylogenetic relationships.

Their somewhat peculiar morphology, which is strikingly similar to that of birds, has caused extensive debate regarding their place on the tree of life^1,2,4–12,16,17,19,29–38^. Early studies suggested an ornithomimid affinity for alvarezsaurs was proposed^35,36,39^, but they are now generally thought to be basal maniraptorans^6–12,40–44^. The origin of alvarezsaurs has also been much discussed. It was initially suggested that alvarezsaurs originated in South America^3–5,27^, but recent discoveries of basal alvarezsaurs in China support an Asian origin of this clade^6,9,11^.

**Figure 1:**
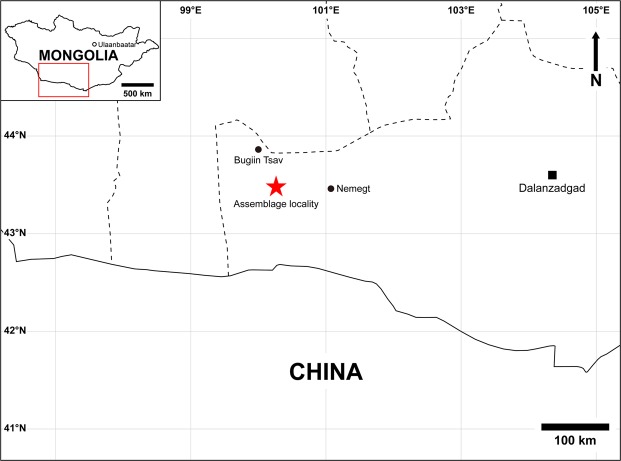
Map showing the locality (marked with a red star) where the multi-species assemblage was discovered.

**Figure 2:**
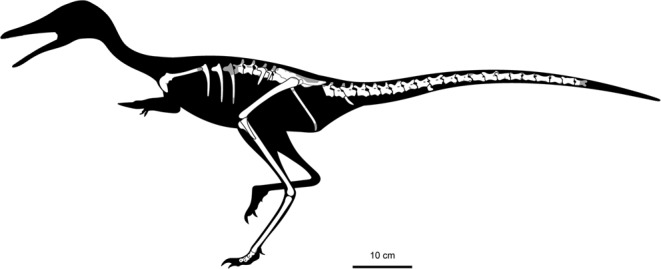
Skeletal reconstruction of *Nemegtonykus citus* gen. et sp. nov. (MPC-D 100/203) with missing parts in grey.

**Figure 3:**
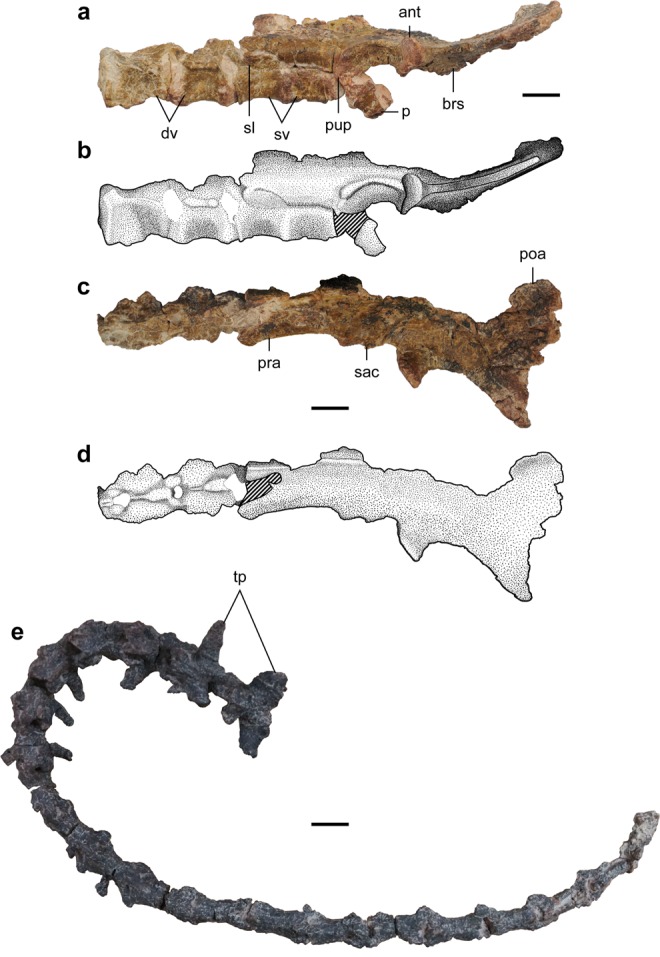
Axial skeleton and pelvis of the holotype specimen of *Nemegtonykus citus* gen. et sp. nov. (MPC-D 100/203). (**a,b**) Photograph (**a**) and interpretative illustration (**b**) of the dorsal-sacral succession and pelvis in left lateral view. (**c,d**) Photograph (**c**) and interpretative illustration (**d**) of the dorsal-sacral succession and pelvis in dorsal view. (**e**) Caudal vertebrae in dorsal view. Abbreviations: ant, antitrochanter; brs, brevis shelf; dv, dorsal vertebra(e); p, pubis; poa, postacetabular process; pra, preacetabular process; pup, pubic peduncle; sac, supracetabular crest; sl, sacral lamina; sv, sacral vertebra(e); tp, transverse process(es). Scale bars equal 1 cm.

**Figure 4:**
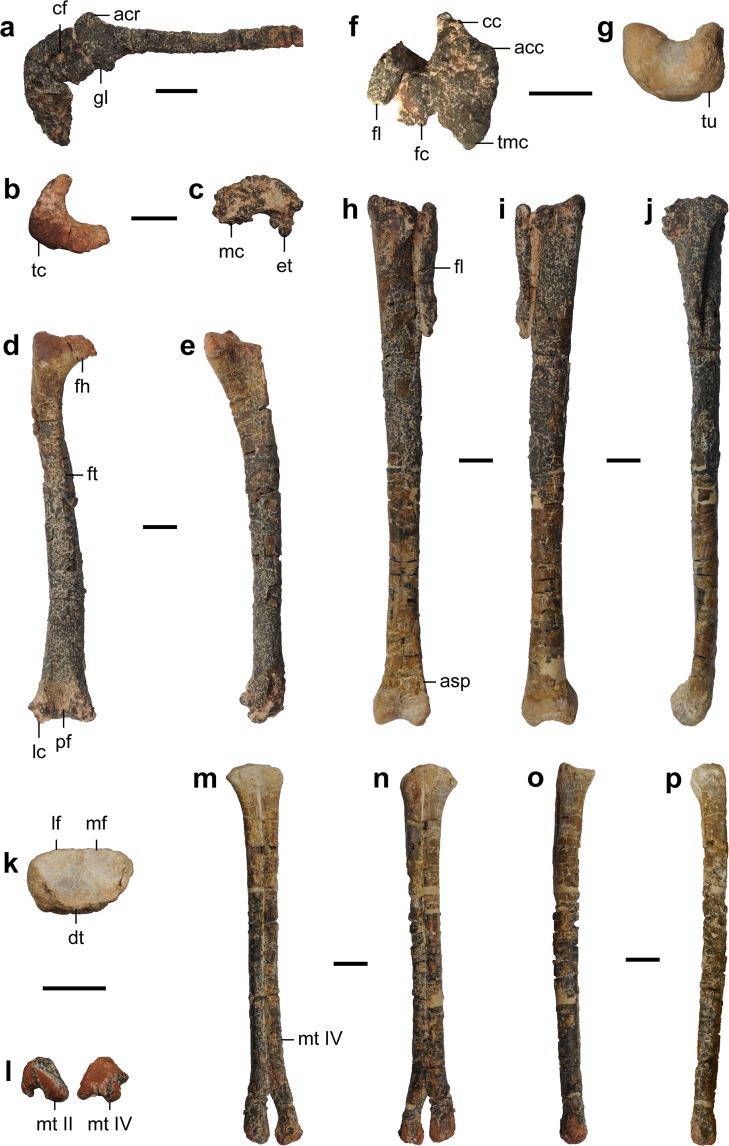
Appendicular skeleton of the holotype specimen of *Nemegtonykus citus* gen. et sp. nov. (MPC-D 100/203). (**a**) Left scapulocoracoid in lateral view. (**b–e**) Left femur in proximal (**b**), distal (**c**), posterior (**d**), and medial (**e**) views. (**f–j**) Left tibiotarsus in proximal (**f**), distal (**g**), anterior (**h**), posterior (**i**), and lateral (**j**) views. (**k–p**) Left tarsometatarsus in proximal (**k**), distal (**l)**, dorsal (**m**), plantar (**n**), medial (**o**), and lateral (**p**) views. Abbreviations: acc, accessory condyle; acr, acromion process; asp, ascending process of astragalus; cc, cnemial crest; cf, coracoid foramen; dt, distal tarsal; et, ectocondylar tuber; fc, fibular condyle; fh, femoral head; fl, fibula; ft, fourth trochanter; gl, glenoid fossa; lc, lateral distal condyle of femur; lf, lateral fossa; mc, medial distal condyle of femur; mf, medial fossa; mt II, metatarsal II; mt IV, metatarsal IV; pf, popliteal fossa; tc, trochanteric crest; tmc, medial proximal condyle of tibiotarsus; tu, tubercle. Scale bars equal 1 cm.

**Figure 5:**
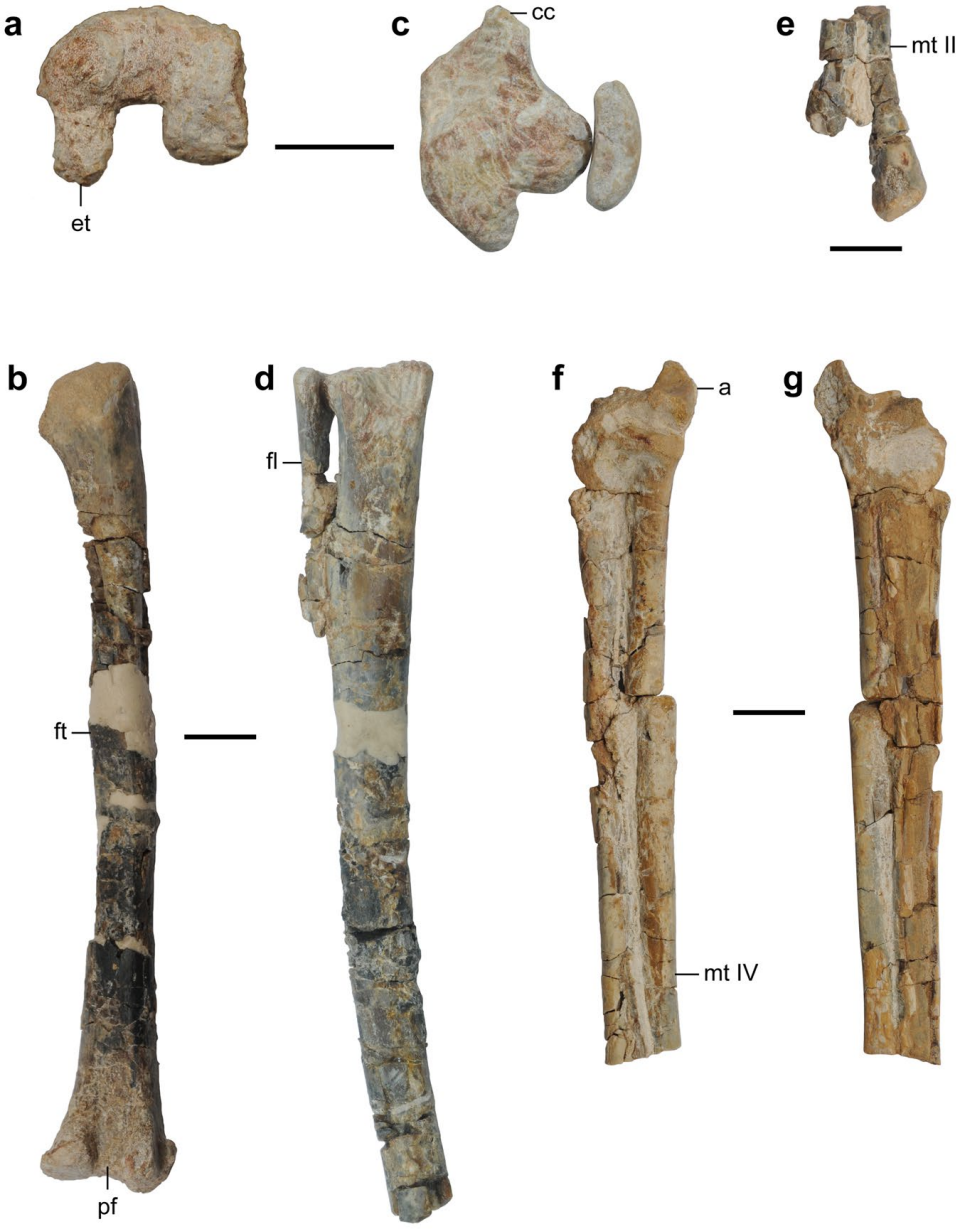
Selected elements of MPC-D 100/207 (referred specimen of *Nemegtonykus citus*). (**a,b**) Right femur in distal (**a**) and posterior (**b**) views. (**c,d**) Right tibia and fibula in proximal (**c**) and anterior (**d**) views. (**e**) Right metatarsals II and IV in dorsal view. (**f,g**) Left astragalus and metatarsus in dorsal (**f**) and plantar (**g**) views. Abbreviations: a, astragalus; cc, cnemial crest; et, ectocondylar tuber; fl, fibula; ft, fourth trochanter; mt II, metatarsal II; mt IV, metatarsal IV; pf, popliteal fossa. Scale bars equal 1 cm.

**Figure 6:**
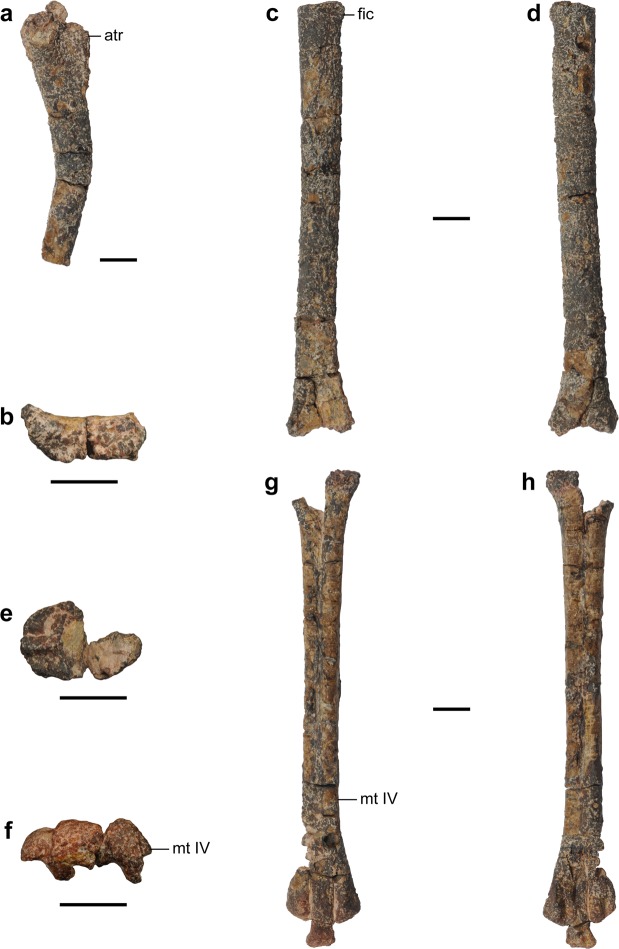
Selected elements of cf. *Mononykus* sp. (MPC-D 100/206). (**a**) Left femur in medial view. (**b–d**) Left tibia in distal (**b**), anterior (**c**), and posterior (**d**) views. (**e–h**) Left metatarsus in proximal (**e**), distal (**f**), dorsal (**g**), and plantar (**h**) views. Abbreviations: atr, anterior trochanter; fic, fibular crest; mt IV, metatarsal IV. Scale bars equal 1 cm.

**Figure 7:**
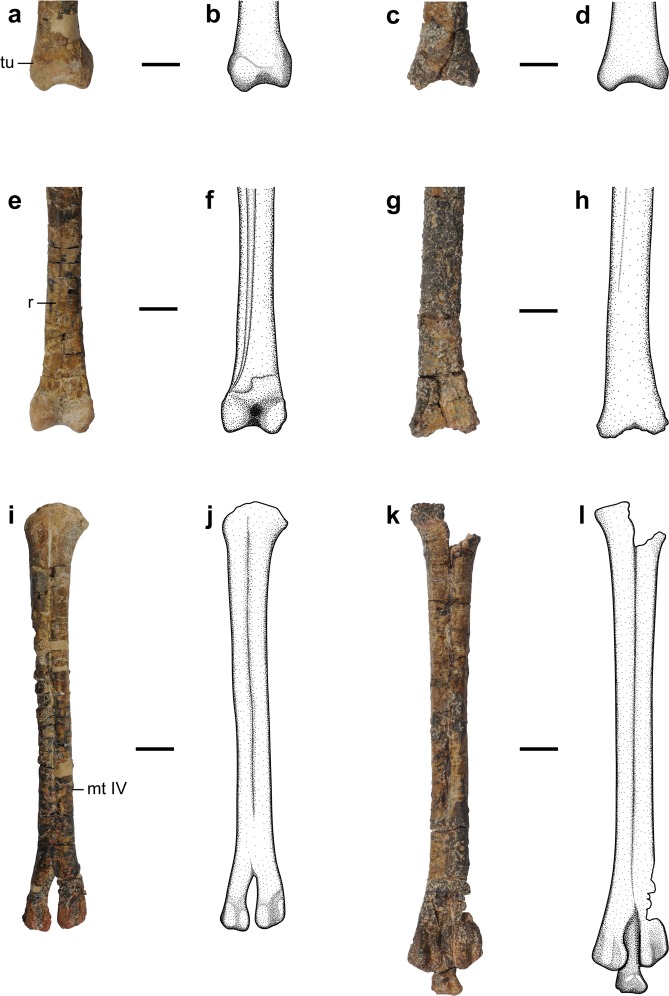
Selected elements and their interpretative illustrations of the holotype specimen of *Nemegtonykus citus* (MPC-D 100/203) and cf. *Mononykus* sp. (MPC-D 100/206) for comparison. (**a–d**) Distal end of left tibiotarsus of MPC-D 100/203 (**a,b**) and distal end of left tibia of MPC-D 100/206 (**c,d**) in posterior views. (**e–h**) Distal part of left tibiotarsus of MPC-D 100/203 (e, f) and distal part of left tibia of MPC-D 100/206 (**g,h**) in anterior views. (**i–l**) Left tarsometatarsus of MPC-D 100/203 (**i,j)** and left metatarsus of MPC-D 100/206 (**k,l**) in plantar views. Abbreviations: mt IV, metatarsal IV; r, ridge; tu, tubercle. Scale bars equal 1 cm.

The Nemegt Basin in the Gobi Desert, which holds three major Upper Cretaceous sedimentary formations (Nemegt, Baruungoyot, and Djadochta), is home to many alvarezsaurids, specifically parvicursorines. However, there has been only one alvarezsaurid taxon, *Mononykus olecranus*, known from the Nemegt Formation^1^. In fact, alvarezsaurid specimens are relatively rare in the Nemegt Formation compared to the older Baruungoyot and Djadochta formations, each of which has yielded two alvarezsaurid taxa^4,15–17,19^. In 2008, an international team of the Korea-Mongolia International Dinosaur Expedition (KID) found a small assemblage (the surface area of the region containing the assemblage is less than 0.5 m^2^) of theropods comprising multiple individuals of oviraptorids and alvarezsaurids from the Nemegt Formation at Altan Uul III in the Gobi Desert, Mongolia (Figs 1 and S1). The site is located on a gentle slope, and many of the fossils were buried inside the rock. The oviraptorid specimens consist of the holotype of *Gobiraptor minutus* and at least two other larger undescribed individuals mainly represented by a sacrum and partial hind limb elements^45^. Similarly, alvarezsaurid specimens in this assemblage include postcranial elements of three individuals which share peculiar hind limb morphology of alvarezsaurids, such as the elongated distal hind limbs, greatly reduced fibula, and the unique arctometatarsal condition. (Figs 2–7 and S2–4). All three alvarezsaurid specimens are described in this paper, including the holotype and referred specimens of a new taxon *Nemegtonykus citus* gen.et sp. nov. The other specimen is distinguished from *Nemegtonykus* but more similar to *M. olecranus*, thus referred to cf. *Mononykus* sp. Multiple alvarezsaurid specimens in this assemblage indicate that alvarezsaurids were more abundant than the previous fossil record suggested during the Nemegt time.

## Results

### Systematic palaeontology

Dinosauria Owen, 1842^46^

Theropoda Marsh, 1881^47^

Maniraptora Gauthier, 1986^48^

Alvarezsauria Bonaparte, 1991^23^

Alvarezsauridae Bonaparte, 1991^23^

Parvicursorinae Karhu and Rautian, 1996^15^

*Nemegtonykus citus*, gen. et sp. nov.

### Holotype

MPC-D 100/203, partially disarticulated postcranial elements including six dorsal vertebrae, two sacral vertebrae, 21 caudal vertebrae, five separated dorsal ribs, nearly complete left scapulocoracoid, nearly complete left ilium, partial right ilium, partial left pubis, other partial pelvic elements, complete left femur, complete left tibiotarsus, partial right tibia, nearly complete left fibula, left tarsometatarsus consisting of distal tarsal co-ossified with metatarsals II and IV, and isolated left pedal phalanges III-1, IV-1, and IV-2 as well as possible II-1 and II-2. Many of the elements such as the sacrum, caudal vertebrae, and left hind limb were naturally articulated and close to each other. The other elements were not articulated but very close to the articulated bones. The exceptions are the dorsal ribs which were in contact with the pelvic elements of MPC-D 102/111 (*Gobiraptor minutus* holotype). The ribs are assigned to MPC-D 100/203 rather than MPC-D 102/111 because of their morphology and size. Their tuberculum and capitulum are arranged to fit the diapophysis and parapophysis which, in case of alvarezsaurids, are located on the same horizontal plane. They are also too small to be a part of MPC-D 102/111 but perfectly match the dorsal vertebrae of MPC-D 100/203.

### Referred specimen

MPC-D 100/207, possible pelvic element, nearly complete right femur, partial right tibia which is articulated with the fibula, partial left astragalus with tarsometatarsus, distal ends of right metatarsals II, III, and IV, and possible right pedal phalanges II-1 and IV-1. The elements were disarticulated but located close to each other with matching sizes. They also exhibit the same light-coloured texture that makes them distinguishable from other specimens.

### Locality and horizon

Altan Uul III^49–53^, Ömnögovi Province, Mongolia (Figs 1 and S1). Upper Cretaceous Nemegt Formation^49–51,53–55^.

### Etymology

The generic name refers to the Nemegt Formation (the origin of the holotype specimen) and onyx (‘claw’ in Greek); specific name means ‘swift’ in Latin, referring to the hypothesized cursorial lifestyle of this taxon.

### Diagnosis

An alvarezsaurid of moderate size distinguished from other alvarezsaurids by the following unique set of characters (autapomorphies are marked with an asterisk, see Supplementary Information for differential diagnosis): completely co-ossified first and second sacral vertebrae; anteroposteriorly elongate and subtrapezoidal lamina formed by transverse process-sacral rib complex and postzygapophyses on the first sacral vertebra, which is co-ossified with preacetabular part of ilium* (similar structure present in *Xixianykus*); co-ossification between the second sacral centrum and preacetabular part of ilium without any contribution of sacral ribs*; partially co-ossified scapulocoracoid*; greatly reduced pubic peduncle (similar to *Qiupanykus*); posterodorsally oriented postacetabular process of ilium*; distinct fossa on dorsal surface of ilium near antitrochanter (also present in *Xixianykus*); prominent wedge-shaped tubercle on the posterolateral margin of tibiotarsus near its distal end*; co-ossification between distal tarsal and metatarsus (also present in *Xixianykus* and *Albinykus*); and partial plantar co-ossification between distal shafts of metatarsals II and IV*.

### Description

The holotype specimen of *Nemegtonykus citus* (MPC-D 100/203) is slightly smaller than that of *Mononykus*^1,2^, but larger than the specimens of *Parvicursor*^15^ or *Shuvuuia*^4,17^. Based on the equation of Campione *et al*.^56^, which involves the mid-shaft circumference of the femur, the estimated body weight of MPC-D 100/203 is approximately 3.4 kg (see Supplementary Table S1 for measurements). The size of the referred specimen (MPC-D 100/207) is comparable to the holotype (Table S2). The full description of MPC-D 100/203 is included in Supplementary Information.

The preserved dorsal centra are opisthocoelous and lack a hyposphene-hypantrum articulation or pleurocoels (Figs 3a–d and S2). They are anteroposteriorly short and laterally compressed with a distinct ventral keel. As in other alvarezsaurids, the parapophyses are located at almost the same level as the diapophyses^4,5,10^. The prezygapophyses are short and anterodorsally oriented. On the dorsal surface of the neural arch of each dorsal vertebra is a sharp ridge that posteriorly leads to the neural spine. The last and penultimate dorsal vertebrae and two anteriormost sacral vertebrae are articulated, forming a dorsal-sacral succession (Fig. 3a–d). The co-ossified first and second sacral centra indicate that a synsacrum was present in life. Like the dorsal vertebrae, the prezygapophyses of the first sacral vertebra are short. Posterior to the prezygapophyses is a dorsolaterally oriented subtrapezoidal lamina which is formed by the transverse process-sacral rib complex and postzygapophyses. This lamina is dorsoventrally tall at its anterior end in contrast to the short posterior end with a gradual decrease in height. It is also possible that the lamina was co-ossified with the preacetabular process of the ilium although most of the contact between them is damaged. The second sacral vertebra completely lacks this lamina or zygapophyses. Its centrum is directly co-ossified with the ilium, which is unique among alvarezsaurids. The preserved caudal vertebrae (Fig. 3e) are articulated with each other, but their exact position in the vertebral column is uncertain. The caudal centra are all procoelous and become proportionally elongate. There is no sign of co-ossification between them. The zygapophyses are short, and the transverse processes are anteriorly located in the proximal caudal vertebrae as in other alvarezsaurids^4,10^. No neural spines are completely preserved, but they become low ridges in the more distal caudal vertebrae. The proximalmost caudal vertebra is distinguished from others, having a posteroventrally oblique anterior articulation surface and mediolaterally short transverse processes. The preserved dorsal ribs (Fig. S2) are not articulated with the vertebrae and lack uncinate processes. The chevrons are mostly fragmentary except for the two complete ones (Fig. S2). One is a proximal chevron and proximodistally elongate. The other is from a more distal position and L-shaped.

The scapulocoracoid is partially co-ossified (Fig. 4a). It has a long blade with a dorsally oriented small acromion process which has a round anterior margin unlike in *Mononykus*^2^. The thin coracoid ventrally tapers to produce a pointed end, and the glenoid fossa faces ventrolaterally.

The ilium is medially inclined, and the vertical surface at its medial margin suggests the two ilia and sacral neural spines met at the middle (Fig. 3a–d). It exhibits a greatly reduced pubic peduncle which is merely a small protrusion, similarly to that of *Qiupanykus*^14^. As in other alvarezsaurids, the antitrochanter is pronounced and horizontal^4,8^. The postacetabular process has a lateral expansion and posterodorsally directed. A small fragment of the pubis is articulated with the ilium. It exhibits an opisthopubic condition which is also known in other alvarezsaurids^2–4,8,15,31^.

The femur of MPC-D 100/203 (holotype) has a medially directed head which is separated from the trochanteric crest (Fig. 4b–e). Its shaft is only slightly bowed anteriorly and has a ridge-like fourth trochanter. On the posterior surface of the distal quarter of the femur, a large popliteal fossa is present. The distal condyles are damaged, but it is clear that a distinct ectocondylar tuber is developed on the posterior surface of the lateral condyle which also has a small lateral projection. Between the two condyles, the lateral one extends distally further than the medial one. In MPC-D 100/207, the femur is missing its head, but the trochanteric crest is intact (Fig. 5a,b). It has a fourth trochanter which is slightly more prominent than that of the holotype. The popliteal fossa is distally open unlike in *Mononykus*^2^ or *Xixianykus*^8^, but similarly to *Parvicursor*^4^ or *Linhenykus*^10^. The lateral distal condyle is less pronounced than the medial one and has an ectocondylar tuber which projects posteriorly beyond the level of the medial condyle. Lateral to the lateral distal condyle is a small tubercle which is confluent with the round distal projection.

The tibia and astragalocalcaneum are largely co-ossified to form a tibiotarsus in the holotype (Fig. 4f–j). It has a cnemial crest and three condyles at the proximal end. Medial to the cnemial crest is an accessory condyle which forms a distinct fossa with the former. The medial condyle is located posteriorly to the accessory condyle and proximally elevated. It also has a proximodistally elongated base on the posterior surface of the tibiotarsus. The fibular condyle, which is articulated with the fibula, is rectangular in proximal view and separated from the medial condyle by a deep notch. Although it is proximodistally expanded in posterior view, the extent is not as significant as in the case of the medial condyle. Distal to the fibular condyle, there is a weak fibular crest that is about 22 mm long. The tibial shaft is straight and bears a low ridge on the posterior surface of its distal half. This ridge is medially placed and becomes a sharp medial margin at the distal end. On the posterior surface near the distal end where a postfibular flange is present in other theropods, a wedge-like posterolateral tubercle is developed, which is unique among alvarezsaurids. The thin ascending process of the astragalocalcaneum has a prominent notch located at the middle as in other parvicursorines^4,8,10^. Consequently, it only covers the lateral half of the anterior surface of the tibiotarsus although how proximally it extends is not certain. There is also a circular and deep fossa at the base of the ascending process. The medial distal condyle is more robust than the lateral one. In MPC-D 100/207, the tibia is longer than the femur although its distal end is not preserved (Fig. 5c,d). The conical medial condyle is proximally elevated. Lateral to the medial condyle is the fibular condyle which is separated from the former by a wide cleft. They lack a proximodistally expanded posterior base present in the holotype, and the fibular condyle has a relatively posterior position in proximal view. The tibial shaft is laterally bowed and has a shallow anteromedial ridge, but it is not certain whether it reaches the distal end. The astragalus articulated with the metatarsus suggests that the tibia is not co-ossified with proximal tarsals.

The fibula of the holotype is proximodistally short, not reaching even the mid-shaft of the tibiotarsus (Fig. 4f,h–j). Its proximal surface is concave, and its shaft tapers distally. There is a lateral crest for the attachment of the M. iliofibularis on the distal third of the shaft. This crest is less prominent than the one in *Albinykus*^20^.

The distal tarsal is co-ossified with metatarsals II and IV forming a tarsometatarsus in the holotype (Fig. 4k–p). Metatarsals II and IV are subequal in proximodistal length, but the latter is slightly longer than the former. The proximal end of metatarsal II is greatly deflected in the medial direction in contrast to the nearly straight one of metatarsal IV. Their shafts tightly adhere to each other along most of their lengths, and the proximal and distal ends of the contact between them show some degree of co-ossification. Although the co-ossification between the distal shafts of metatarsals II and IV is limited to their plantar surface, this feature is not known in any other alvarezsaurids. The distal condyles of metatarsals II and IV are comparable in size and non-ginglymoid. In MPC-D 100/207, metatarsals are heavily damaged and partially preserved (Figs 5e–g and S3). It is not clear if the metatarsus is co-ossified with distal tarsals. Proximally, metatarsal II is much wider than metatarsal IV in mediolateral width in plantar view, and this dissimilarity in width decreases distally. The proximal end of metatarsal IV shows a subtle lateral deflection as in the holotype. A prominent plantar flange is present on the shaft of metatarsal IV, which is more distinct than that of the holotype or MPC-D 100/206.

The pedal phalanges of the holotype are generally short and partially preserved (Fig. S2). An extensor fossa is present in most of the preserved pedal phalanges. The distal articular ends are distinct and large. The two phalanges of MPC-D 100/207 are possibly right pedal phalanges II-1 and IV-1 (Fig. S3). Possible phalanx II-1 is proximodistally long and proximally robust compared to the slender distal end. The deep proximal articulation surface is subcircular. A deep extensor fossa is present on the dorsal surface near the distal articular hemicondyles. On the plantar surface opposite to the extensor fossa is a depression which could be a flexor fossa which is also known in *Linhenykus*^10^. The lateral hemicondyle has a massive plantar expansion and is larger than the medial one. Possible phalanx IV-1 is proximodistally short but robust as in other parvicursorines^2,10^. It is stout and has a deep extensor fossa which is comparable in size to that of possible phalanx II-1. On the other hand, the flexor fossa of this phalanx is relatively small. The circular collateral ligament fossa is well developed on the medial hemicondyle.

Dinosauria Owen, 1842^46^

Theropoda Marsh, 1881^47^

Maniraptora Gauthier, 1986^48^

Alvarezsauria Bonaparte, 1991^23^

Alvarezsauridae Bonaparte, 1991^23^

Parvicursorinae Karhu and Rautian, 1996^15^

cf. *Mononykus* sp.

### Material

MPC-D 100/206, seven caudal vertebrae and left hind limb and pes including partial left femur, partial left tibia, distal tarsal, nearly complete left metatarsals II, III, and IV, and left pedal phalanges IV-1 and IV-2. The left hind limb was nearly articulated *in situ* and close to the partially articulated caudal vertebrae, which are slightly larger than those of MPC-D 100/203.

### Locality and horizon

Same as MPC-D 100/203 and MPC-D 100/206.

### Description

The elements of MPC-D 100/206 are slightly larger than those of the holotype specimen of *Nemegtonykus citus* (MPC-D 100/203) (see Supplementary Table S3 for measurements). MPC-D 100/206 is referred to cf. *Mononykus* sp. based on their many similarities. The full description of MPC-D 100/206 is included in Supplementary Information.

The preserved caudal vertebrae are all procoelous and lack pleurocoels (Fig. S4). The proximal centra do not have a distinct ventral furrow unlike in *Nemegtonykus*. The zygapophyses are short, and the neural spine appears to be just a low ridge in more distal caudal vertebrae.

The femur is missing its distal half as well as its head region (Fig. 6a). The shaft is strongly bowed in the anterior direction, differing from that of *Nemegtonykus*. Instead, its prominent curvature recalls the femoral shafts of *M. olecranus*^2^ or *Qiupanykus*^14^. A ridge-like fourth trochanter is present on the posteromedial margin of the proximal shaft.

The tibia is represented by its shaft and distal end (Fig. 6b–d). As in *Nemegtonykus* or *M. olecranus*^2^, the tibial shaft is straight. It also has a very shallow and short ridge on the anteromedial surface. It is not connected with the medial margin of the distal end, unlike in *Nemegtonykus*. The posterior surface of the distal end is flat and lacks any tubercle, which is also different from *Nemegtonykus*. The tibia is completely separated from the proximal tarsals. This condition is dissimilar to *Nemegtonykus* or *M. olecranus* where they are partially co-ossified^2^.

The metatarsus has a typical parvicursorine arctometatarsal condition (Fig. 6e–h). It is not co-ossified with a distal tarsal which is a thin plate-like bone (Fig. S4). Proximally, metatarsals II and IV are medially and laterally deflected to a great extent, respectively as in *M. olecranus*^2^. There is no sign of co-ossification between them in any region. Their distal trochleae are similar in morphology to those of other parvicursorines^2,4,10,15^. Metatarsal III has a pair of deep collateral ligament fossae at its distal end.

### Phylogenetic analysis

**Figure 8:**
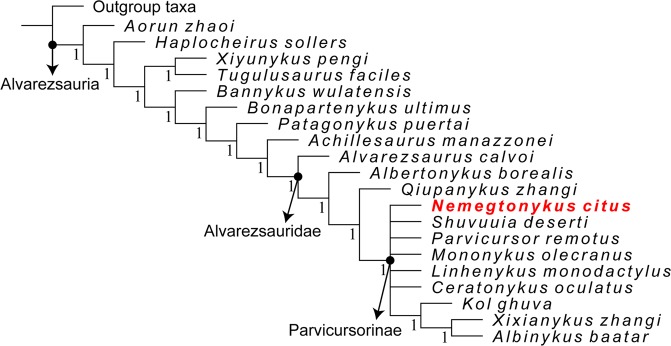
Phylogeny of Alvarezsauria on the strict consensus tree. Numbers at each node indicate Bremer support values.

The phylogenetic relationships among alvarezsaurs as recovered by the strict consensus tree (Figs 8 and S5) are very similar to the result of Xu *et al*.^11^ with only slight differences. In other words, the addition of *Qiupanykus* and *Nemegtonykus* into the data matrix did not significantly affect the overall tree topology of Alvarezsauria. The most notable difference is the inclusion of *Kol*, *Xixianykus*, and *Albinykus* in the subfamily Parvicursorinae. The phylogenetic analysis of Xu *et al*.^11^ placed these three taxa outside Parvicursorinae along with *Albertonykus* which was also recovered as a non-parvicursorine alvarezsaurid on the strict consensus tree in this study. Among the non-parvicursorine alvarezsaurids, *Alvarezsaurus* is located at the basalmost position. On the other hand, *Qiupanykus* was recovered as a sister taxon to Parvicursorinae. Our analysis also shows that *Nemegtonykus* is a parvicursorine and forms a polytomy with *Shuvuuia*, *Parvicursor*, *Mononykus*, *Linhenykus*, and *Ceratonykus*, all of which are from Upper Cretaceous deposits in the Gobi Desert.

## Discussion

According to the result of our phylogenetic analysis, members of Parvicursorinae share the following synapomorphies (see Supplementary Fig. S6 for the list of common synapomorphies): smooth ventral surface of anterior cervicals (263:1), anterior cervical centra extending beyond the posterior end of neural arch (267:1), ventral surface of first sacral centrum mediolaterally constricted with a keel (313:1), anteriorly displaced transverse processes of proximal caudals (330:1), closed popliteal fossa on the distal end of femur (528:1), and astragalus and calcaneum fused to each other and to tibia (557:1). The phylogenetic relationships among parvicursorines are not fully resolved, but it is worth noting that all known parvicursorines are geographically very close except for *Xixianykus*. This highlights the importance of the Gobi Desert area for its documentation of parvicursorine diversification events in the Late Cretaceous. Nevertheless, the poor resolution of Parvicursorinae calls for more complete specimens in order to clarify the relationships among parvicursorines.

Although all three alvarezsaurid specimens in this study were associated with each other, they exhibit several morphological differences, especially between MPC-D 100/203 (*Nemegtonykus* holotype) and MPC-D 100/206 (Fig. 7). MPC-D 100/206 is distinguished from *Nemegtonykus* by the following characters: absence of a distinct furrow on the ventral surface of proximal caudal centra, a much reduced ridge on the anteromedial surface of the tibia, a lack of a tubercle on the distolateral margin of tibia, and a strong lateral deflection in the proximal end of metatarsal IV. However, most of these characters of MPC-D 100/206 are shared with *M. olecranus*, especially those of the tibia and metatarsus^2^. The only notable difference between *M. olecranus* and MPC-D 100/206 is the extent of co-ossification of tibia with proximal tarsals, which could be related to ontogeny. Because MPC-D 100/206 has missing elements which could have diagnostic characters of *M. olecranus*, it is identified as cf. *Mononykus* sp.

The Nemegt Formation is one of the most productive sedimentary formations in terms of dinosaur fossils, but alvarezsaurid specimens have been extremely rare. There has been only one known taxon, *M. olecranus* which is represented by an incomplete skeleton. In contrast, the older Baruungoyot and Djadochta formations, both of which are closely located to the Nemegt Formation, have so far yielded a total of four alvarezsaurid taxa with multiple specimens referred to *Shuvuuia*^4,17^. Further, there has been no record of new alvarezsaurid specimens from the Nemegt Formation since the initial report of *Mononykus*^1^. The discovery of the three additional alvarezsaurid specimens described here is thus important because it tells us that alvarezsaurids were more abundant and diverse in the Nemegt fauna than previous evidence suggested. The scarcity of alvarezsaurid materials in the Nemegt Formation may be, therefore, related to preservational biases rather than actual diversity or abundance of this family. Moreover, the increased diversity of alvarezsaurids in the Nemegt Formation indicates that they were successfully adapted to a wet environment as well as dry environments represented by the Baruungoyot and Djadochta formations^50,53,57–61^. In addition, their diversity in the Nemegt Basin suggests that parvicursorines must have constantly diversified during this time period.

## Methods

### Phylogenetic analysis

For a new analysis, we slightly modified the terminal taxa, character lists, and data matrix from the dataset of Xu *et al*.^11^ (see Supplementary Information for the character modification and data matrix). Two additional taxa (*Qiupanykus zhangi* and *Nemegtonykus citus*) are included in the data matrix which incorporates a total of 115 taxa with 594 characters. The data matrix was then analysed by using TNT version 1.5^62^. A traditional search (Wagner trees with 1000 random seeds and 1000 random-addition-sequence replications, Tree bisection and reconnection (TBR) algorithm for the swapping algorithm, and 10 trees to save per replication) was implemented and resulted in 500 most parsimonious trees with 3226 steps with consistency index (CI) of 0.217 and retention index (RI) of 0.604. A strict consensus tree (Fig. S6) was then generated, and the Bremer support values for each node of the strict consensus tree was calculated by utilization of the ‘Bremer.run’ script provided by TNT^62^. Adobe Illustrator CC 2018 was employed to produce the image of the phylogenetic tree for publication.

## Supplementary information

Supplementary Information
