# Supplementary material for: A new alvarezsaurid dinosaur from the Nemegt Formation of Mongolia
Source: Sci Rep. 2019 Oct 29;9:15493. doi: 10.1038/s41598-019-52021-y (PMC6820876; doi:10.1038/s41598-019-52021-y)
Supplement: Supplementary file 1 — Supplementary Information [file 41598_2019_52021_MOESM1_ESM.docx]

A new alvarezsaurid dinosaur from the Nemegt Formation of Mongolia

Sungjin Lee, Jin-Young Park, Yuong-Nam Lee, Su-Hwan Kim, Junchang Lü, Rinchen Barsbold, and Khishigjav Tsogtbaatar

1. Differential diagnosis for *Nemegtonykus citus*

2. Description of MPC-D 100/203

3. Description of MPC-D 100/206

4. Supplementary Figures

5. Supplementary Tables

6. Character modification statement

7. Data matrix

8. Supplementary References

**1. Differential diagnosis for *Nemegtonykus citus***

Different from *Mononykus* *olecranus*^1,2^ in: partial co-ossification between scapula and coracoid, anterior margin of acromion process of scapula round with centrally located peak (vertical anterior margin with anteriorly located peak in *Mononykus*), convex lateral surface of scapula above glenoid (concave in *Mononykus*), pubic peduncle of ilium more reduced and knob-like, tibiotarsus bearing tubercle on posterolateral margin near distal end, co-ossified distal tarsal and metatarsus, and no collateral ligament fossa on distal articular ends of metatarsals II and IV.

Different from *Shuvuuia deserti*^3-5^ in: partially co-ossified scapula and coracoid, co-ossification between first sacral vertebra and preacetabular process of ilium by subtrapezoidal lamina, second sacral centrum directly co-ossified with ilium, postacetabular process of ilium more dorsally oriented, tibial shaft straight, co-ossified tibia and astragalocalcaneum forming tibiotarsus, deeper excavation at medial margin of ascending process of astragalus, ascending process of astragalus rising from medial end of medial condyle, thin spine at the distal end of fibula absent, and co-ossified distal tarsal and metatarsus.

Different from *Parvicursor remotus*^6^ in: posterodorsally extending postacetabular process of ilium, less bowed femoral shaft, weak fourth trochanter of femur (absent in *Parvicursor*), straight shaft of tibiotarsus, tibiotarsus with distally developed tubercle on posterolateral margin, deeply excavated medial margin of ascending process of astragalus, and co-ossification between distal tarsal and metatarsus.

Different from *Ceratonykus oculatus*^7^ in: thin coracoid, absence of any crest on the dorsal surface of postacetabular process of ilium, less bowed femoral shaft, straight shaft of tibiotarsus, presence of tubercle on the posterolateral margin of tibiotarsus near its distal end, and partial plantar co-ossification between distal shafts of metatarsals II and IV.

Different from *Kol ghuva*^8^ in: distal tarsal and metatarsus co-ossified together, proximal end of metatarsal II medially deflected, proximal end of metatarsal II mediolaterally wider than that of metatarsal IV, proximodistal lengths of metatarsals II and IV subequal, partial co-ossification between distal shafts of metatarsals II and IV, and presence of lateral flange on distal end of metatarsal IV.

Different from *Albinykus baatar*^9^ in: shaft of tibiotarsus straight, prominent tubercle on posterolateral margin of distal tibiotarsus, no groove on ascending process of astragalus, crest on lateral surface of fibula for attachment of M. iliofibularis proportionally less developed, shafts of metatarsals II and IV not bowed, and distal shafts of metatarsals II and IV partially co-ossified.

Different from *Xixianykus* *zhangi*^10^ in: second sacral centrum directly co-ossified with ilium without any lamina, posterodorsally oriented postacetabular process of ilium, pubic peduncle of ilium greatly reduced, femoral shaft less curved, oblique proximal surface of tibiotarsus due to elevated medial condyle, no lateral tuber on lateral condyle of distal femur, ectocondylar tuber of femur less prominent, distal tibial shaft lacking lateral step, presence of tubercle on posterolateral surface of distal tibiotarsus, and straight fibular shaft.

Different from *Linhenykus monodactylus*^11,12^ in: subcircular articular surfaces of dorsal centra (subtriangular in *Linhenykus*), opisthocoelous penultimate dorsal centrum (biconvex in *Linhenykus*), convex anterior articular surface of last dorsal centrum (concave in *Linhenykus*), less prominent ventral keel of last dorsal centrum, no ventral groove on either first or second sacral centrum, prominently developed glenoid lip, lateral ridge bordering popliteal fossa on posterior surface of distal femur being more prominent than medial ridge (reverse in *Linhenykus*), no accessory crest on fibular condyle of tibiotarsus, tibiotarsus with tubercle on posterolateral margin near distal end, co-ossified distal tarsal and metatarsus, and partially co-ossified distal shafts of metatarsals II and IV.

**2. Description of MPC-D 100/203**

The holotype specimen of *Nemegtonykus citus* (MPC-D 100/203) consists of postcranial elements which appear to be largely dark-coloured except for a few dorsal vertebrae (Figs 2–4, S2).

**Axial skeleton.** The preserved vertebrae consist of four partial dorsal vertebrae (Fig. S2), two nearly complete posterior-most dorsal vertebrae which are articulated with two anterior-most sacral vertebrae (Fig. 3a–d), and 21 caudal vertebrae, all of which lack neurocentral sutures or pleurocoels (Fig. 3e). There is no hyposphene-hypantrum articulation between any of the preserved vertebrae. It is also absent in other parvicursorines^4,12,13^, but present in *Patagonykus puertai*, which is a basal alvarezsaur^14^. Two of the four partially preserved dorsal vertebrae are isolated and preserved only with neural arches, each of which has a dorsal midline ridge. The other two partial dorsal vertebrae are similar in morphology and articulated with each other. Between these two, the anterior one is missing its anterior half whereas the posterior one is relatively intact. The broken surface of the latter suggests it could be the third last dorsal vertebra. It has a laterally constricted and opisthocoelous centrum forming a prominent keel whose cross-section is thus triangular as in *Mononykus*^2^ or *Linhenykus*^12^. The prezygapophyses are short and anterodorsally oriented. The transverse processes are anteroposteriorly wide at each base and bear diapophyses and parapophyses on the approximately same plane. There is also a well-developed midline ridge on the dorsal surface of the neural arch. This ridge extends posteriorly to the base of the neural spine which is broken off. The short postzygapophyses face ventrolaterally. The centrum of the penultimate dorsal vertebra is also opisthocoelous and anteroposteriorly longer than dorsoventrally high or transversely wide. The posterior articular surface is subcircular, ventrally decreasing in transverse width. The laterally constricted centrum and has a ventral keel as does the possible third last dorsal centrum. The ventral keel is pinched at the middle, having an hourglass shape in ventral view. The neural arch has short zygapophyses and a sharp midline ridge. The last dorsal vertebra has a convex anterior articular surface. The condyle is ball-shaped and fits the concave posterior articular surface of the preceding centrum. The posterior articular surface of the last dorsal centrum is, however, not clearly observable although it seems to be convex. In *Mononykus* and *Shuvuuia*, the last dorsal centrum is biconvex^4^, but in *Linhenykus*, it has a concave anterior articular surface^12^. The last dorsal centrum of *Nemegtonykus* is slightly longer than the preceding centrum in anteroposterior length. It is also dorsoventrally higher than transversely wide. It maintains a laterally compressed condition, but to a lesser degree than that of the penultimate dorsal vertebra. As a result, the ventral keel is not as sharp in the preceding vertebra but it is slightly round. The posterior end of the centrum is transversely much wider than the anterior end. The transverse processes are directed posterolaterally. The zygapophyses are short, and a high midline ridge is present on the dorsal surface of the neural arch. The neural spine of the last dorsal vertebra is broken off as in preceding vertebrae.

The right side of the preserved sacral vertebrae is substantially damaged in contrast to the left side which only exhibits minor breakage. Two preserved sacral centra are co-ossified, and there is no recognizable suture between them. The exact number of the sacrals is uncertain although it is highly unlikely that there were more than seven scarals, considering the anteroposterior lengths of the first and second sacral centra and shape of the ilium. The sacral counts in *Shuvuuia* and *Xixianykus* are seven^4,10^, and four sacral vertebrae are known in *Alvarezsaurus calvoi*^15^ and *Patagonykus puertai*^14^ although they might have had five or more sacrals^4,14,15^. The sacral centra of *Nemegtonykus* show lateral compression as in dorsal centra, but it is not as extensive. Ventrally, the first sacral centrum has a slightly pinched surface, whereas the ventral surface of the second sacral centrum is almost flat. The first sacral centrum is transversely expanded at the anterior end, which corresponds to the similarly enlarged posterior articular surface of the last dorsal centrum. The first and second sacral centra are more or less the same in anteroposterior length as well as in the dorsoventral heights. The prezygapophyses of the first sacral vertebra are short and anterodorsally oriented, similar to those of the dorsal vertebrae. Just posterior to the base of the prezygapophyses, there is a dorsolaterally oriented lamina which is formed by the transverse process-sacral rib complex and postzygapophyses. It might have been co-ossified with the preacetabular part of the ilium. This lamina is comparable to the anterior lamina of *Xixianykus*^10^. It is, however, different from that of *Xixianykus* being dorsolaterally oriented rather than horizontal and present only on the first sacral vertebra. In *Xixianykus*, the three anterior-most sacral vertebrae contribute to the development of the anterior lamina^10^. The lamina of the first sacral vertebra in *Nemegtonykus* is subtrapezoidal in shape, decreasing in dorsoventral height from anterior to posterior direction. It is also anteroposteriorly elongate and reaches the level of the posterior end of the centrum. The second sacral centrum is co-ossified with the preacetabular part of the ilium without any involvement of laminae or sacral ribs, which is unique among alvarezsaurids although the morphology of the synsacrum is not known in many other Mongolian taxa such as *Mononykus*^1,2^, *Parvicursor*^6^, *Ceratonykus*^7^, and *Albinykus*^9^. There are no zygapophyses or sacral ribs on the second sacral vertebra, and the presence of a neural spine is uncertain because of poor preservation. The posterior end of the second sacral centrum is also damaged so the articulation surface is not visible.

The preserved caudal vertebrae include 21 consecutive ones all of which are procoelous. Each vertebra is nearly complete although the most distal one is missing its distal half. In general, the caudals have similar morphology to those of other alvarezsaurids, especially of *Shuvuuia*^4,5^. The exact position of the preserved caudal vertebrae is not certain, but the distinctly different morphology of the proximal-most caudal vertebra from that of the following ones suggests that it is from among the most proximal ones. For convenience, the caudal vertebrae are designated here as caudal A to caudal U, caudal A being the most proximal one and caudal U the most distal one. The anteroposterior lengths of the caudal centra do not exhibit a consistent pattern, but instead they seem to change haphazardly unlike *Alvarezsaurus* whose distal caudal centra are anteroposteriorly longer than proximal ones^4,15^, or *Linhenykus* which has gradually shortened caudal centra^12^. In *Shuvuuia*, the distal caudal centra exhibit a gradually decreasing pattern^5^.

The preserved caudal vertebrae can be divided into three different groups based on their morphology. The first group consists only of caudal A which is distinguished from the rest of the caudals by the posteroventrally inclined anterior articulation surface, presence of a ventral keel instead of a furrow, and the stout transverse processes. The subcircular anterior articular surface is distinctly concave. The centrum of caudal A is laterally compressed, but only to a limited extent. This produces a ventral keel which is reversed V-shaped in lateral view. Posteriorly, the ventral keel mediolaterally widens resulting in a smooth surface. The elliptical neural canal is half the size of the anterior articulation surface at the proximal end and becomes distally smaller. The neural arch pedicles are located anteriorly as in other alvarezsaurids^4,12^, and the base of the transverse processes extends beyond the anterior end of the centrum. The transverse processes of caudal A are anteroposteriorly longer than those of the following caudals but mediolaterally shorter than those of caudal B. Prezygapophyses are missing in caudal A. There is a very short and steep midline ridge on the dorsal surface of caudal A, which leads to the base of the neural spine. The neural spine and postzygapophyses are located at the level of the mid-length of the centrum.

The second group consists of caudals B to J. They are distinguished from the more distal caudals by the presence of transverse processes and relatively high ratios of dorsoventral height to anteroposterior length. The caudal vertebrae in the second group are generally similar, but distally the centra become anteroposteriorly much longer than dorsoventrally high. All of the centra in this group exhibit a small degree of lateral compression having a reversed V-shaped ventral margin in lateral view and a ventral furrow bordered by two longitudinal ridges. The neural arch pedicles are positioned anteriorly in caudals B through D, but transverse processes in these caudals are located more posteriorly than those of caudal A. From caudal E, the position of transverse processes is near the level of mid-centrum due to the posterior positioning of the neural arch pedicles. The transverse processes of the second group are also more gracile than those of caudal A. Caudal B has transverse processes that are the longest in mediolateral length and also perpendicular to the prezygapophyses, whereas those of the rest of the caudal vertebrae are progressively shorter and anteroposteriorly oriented. Transverse processes with a posterolateral orientation are also shown in *Linhenykus*^12^, *Shuvuuia*^4^, and *Alvarezsaurus*^15^, but they are anterolaterally oriented in *Parvicursor*^6^. The long axis of the transverse process is dorsally inclined in all the second group caudal vertebrae. The morphology of zygapophyses in this group is rather conservative with long, medially facing prezygapophyses and short, ventrolaterally facing postzygapophyses. The prezygapophyses become distally shorter and have a anterodorsal orientation as in *Haplocheirus*^16^ as well as other alvarezsaurids^4,12,17^. The dorsal midline ridges are more prominent in this group than in caudal A. It is clear that neural spines become thinner and ridge-like in more distal caudals even in the second group. Consequently, the neural spines are reduced into a low ridge from caudal H.

The third group includes caudals K to U which may belong to the distal part of the tail. They are conspicuously longer anteroposteriorly than dorsoventrally high with greatly reduced neural arches. Including caudal U, each centrum in the third group exhibits lateral compression and a ventral furrow. A ventral furrow in distal caudal vertebrae is also known in alvarezsaurid specimens from Uzbekistan^17^. Distal caudal vertebrae are not well known in alvarezsaurids except for *Shuvuuia* whose caudal vertebrae are slightly different in morphology, having lateral grooves and small neural canals^5^. In the third group, the caudal centra do not have any grooves on the lateral surface, and the neural canal in each vertebra is relatively large. The neural spines become low ridges which are almost inconspicuous. The anterodorsally oriented prezygapophyses are very short and do not extend beyond one-fourth of the length of each preceding centrum unlike in other non-avian theropods. The postzygapophyses of the preserved distal-most caudal vertebrae are greatly reduced in size being almost invisible in dorsal view.

Among the preserved dorsal ribs, two are much shorter than the rest (Fig. S2). The longer ribs are likely from mid-dorsal vertebrae, whereas the shorter ones are possibly from those that are close to the synsacrum judging by their lengths and nearly perpendicular curvature. The dorsal ribs have a pronounced capitulum and a much smaller tuberculum. The shape of the dorsal ribs suggests that parapophyses of mid-dorsals are more ventrally located than the diapophyses. The longer dorsal ribs have a general crescentic curvature, but the two shorter ones are abruptly curved to form a boomerang shape. There is no uncinate process on any preserved ribs, but two of the longer ribs have an elongate depression on the dorsal surface of their shafts.

The two complete chevrons (Fig. S2) are each from between caudals B and C and between caudals I and J. They are similar to those of *Linhenykus*^12^, *Shuvuuia*^4^, and *Alvarezsaurus*^15^ in morphology. The anterior one is proximodistally elongate and posteroventrally oriented. At the proximal end, a bony bridge connects the left and right articular surfaces as in *Linhenykus*^12^. The vertically long hemal canal is just ventral to this bridge. The shaft becomes distally thinner and posteriorly curves a little. The other chevron (mid-caudal chevron) is flat distal to the articular surface. It is also anteroposteriorly elongate, but the proximodorsal length is reduced compared to the anterior chevron. As a result, the mid-caudal chevron is L-shaped with a small anterior protrusion. This is slightly different from the inverted T-shaped distal chevrons of *Shuvuuia*^4,5^ and *Alvarezsaurus*^15^.

**Appendicular skeleton.** The preserved appendicular skeleton includes incomplete pectoral and pelvic girdles and hind limb elements including pedal phalanges, most of which are from the left side (Figs 3, 4, S2).

The scapula and coracoid are co-ossified at the middle, but they are dorsally separated and very tightly attached to each other at their ventral regions (Fig. 4a). It seems that the co-ossification between these two bones started first in the middle and proceeded towards the glenoid fossa. In *Linhenykus*, they are fused at the glenoid region^12^. On the other hand, they are separated from each other in other alvarezsaurs such as *Mononykus*^2^, *Patagonykus*^14^, *Shuvuuia*^4^, and *Alvarezsaurus*^15^. The scapular blade is missing its posterior end, but otherwise it is complete. It is nearly straight like that of *Mononykus*^2^ or *Shuvuuia*^4^ although the dorsal margin has a slight medial deflection at the mid-length. In more basal alvarezsaurs, the scapular blade is distinctly curved in medial direction^15,18^. The scapular blade of *Nemegtonykus* is also mediolaterally compressed and anteriorly thickens resulting in the dorsoventrally expanded anterior end. The acromion process is small having a round anterior margin with the centrally located peak, whereas *Mononykus* has an acromion process with a vertical anterior margin which results in the anteriorly positioned peak^2^. Above the glenoid fossa, the lateral surface of the scapula is convex unlike the concave surface in *Mononykus*^2^. The glenoid fossa is ventrolaterally oriented, and the glenoid lip is well developed as in other parvicursorines^4^ except for *Linhenykus* which has a weak glenoid lip^12^. The coracoid is a crescent-shaped thin plate. It also has a relatively thick region near the scapula. The medial and lateral surfaces of the coracoid are smooth without any ridge. A flat and smooth surface is also seen in the coracoids of *Mononykus*^2^, *Alvarezsaurus*^15^, and *Shuvuuia*^4^. However, in *Ceratonykus*, a small crest is present near the posteroventral margin of the coracoid^7^. Basal alvarezsaurs such as *Patagonykus* or *Bonapartenykus* have a distinct ridge on the lateral surface of the coracoid which is medially curved and has a relatively thick dorsal margin^14,18^. The glenoid lip on the coracoid is broken off in MPC-D 100/203. The small coracoid foramen is subcircular and located at the centre of the coracoidal shaft. Biceps tubercle is absent as in other alvarezsaurids^2,4^. The dorsoventrally elongate and thin ventral blade is triangular in shape, tapering ventrally.

The left ilium (Fig. 3a–d) is co-ossified with the first and second sacral vertebrae. It is low and medially inclined to a large extent being nearly horizontal as in *Parvicursor*^6^, *Shuvuuia*^4^, *Xixianykus*^10^, and perhaps *Linhenykus*^12^. Medially, the left ilium has a flat and vertical surface which would have met the right one. The pre- and postacetabular processes are partially preserved so it is difficult to determine the exact anteroposterior length of the ilium. The preacetabular process ventrally extends to meet the second sacral centrum. Aside from this region, it is flat, extending to a dorsomedial direction. The postacetabular process expands laterally (ventrally if not inclined) towards the posteror end, thereby the lateral margin displays a wide curvature in dorsal view like that of other parvicursorines^4,6,10^. In these taxa, the postacetabular process is also horizontal being on the approximately same level of the antitrochanter in lateral view^4,6,10^. However, in *Nemegtonykus*, it is posterodorsally curved forming a distinct fossa on its dorsal (lateral if not inclined) surface near the antitrochanter. This fossa might have had a function similar to the one for M. iliofibularis in other non-avian theropods accommodating more muscle mass^19^. Although it does not have a dorsally curved postacetabular process, a fossa is also known in *Xixianykus* dorsal to the antitrochanter^10^. On the ventral (medial if not inclined) surface of the postacetabular process, the longitudinal brevis shelf demarcates the shallow brevis fossa. Anterior to the brevis shelf is a medially facing subrectangular fossa that might be an articular facet for the synsacrum, which is also present in *Xixianykus*^10^. The acetabulum is overhung by the partially broken supracetabular crest which posteriorly diminishes and completely disappears near the antitrochanter. This is also the case for *Mononykus*^2^. In *Xixianykus*, the supracetabular crest extends far posteriorly although its anterior region is more prominent^10^. The origin of the supracetabular crest is at the level of the centre of the pubic peduncle as in other parvicursorines^2,10^ but unlike in a more basal alvarezsaur *Patagonykus*, where it is more posteriorly located^14^. Around the acetabulum, there is also a well-developed and concave lateral wall that bridges the pubic and ischiadic peduncles. The pubic peduncle is greatly reduced compared to the anteroposteriorly elongate ones in other alvarezsaurids^2,4,10,12^ except for *Qiupanykus* which has a knob-like small pubic peduncle as well^20^. As in other parvicursorines, the ischiadic peduncle is small and transversely oriented^10^. It is also anteroposteriorly narrow and ridge-like, forming a convex ventral margin. At the posterior end of the acetabulum, a massive antitrochanter is developed in a nearly horizontal orientation. It laterally extends as in other parvicursorines^2,4,10^, and the articular surface for the femur faces anterolaterally like that of *Mononykus*^2^ but differing from *Xixianykus* where it faces anteriorly^10^. The dorsal margin of the antitrochanter is above the level of the supracetabular crest.

The preserved left pubis in articulation with the ilium is a proximal part and slightly offset from the pubic peduncle (Fig. 3a, b). The articular surface for the ilium is not visible and neither is the one for the ischium. At the proximal end, the pubis is anteroposteriorly elongate but soon becomes narrower. The lateral surface is flat and smooth without any fossa. The orientation of the preserved part of the pubis suggests an opisthopubic condition as in other parvicursorines^4^. The isolated pelvic elements (Fig. S2) are possibly pubic shafts. The longer two are rod-like, and the shortest one is laterally compressed. The longest one has a distinct diagonal ridge along its entire longitudinal length. It is subtriangular in cross-section at the possibly proximal end but becomes circular towards the other end as in pubes of *Shuvuuia*^4^ and *Patagonykus*^14^. Another longer one has a progressively compressed shaft which also slightly curves. This morphology is known in distal pubes of other parvicursorines^10^. The shortest one has a longitudinal groove extending from the broader extremity to the narrower shaft indicating that it may be from the proximal pubis^4,10^.

The left femur is completely preserved except for the anterior half of the trochanteric crest (Fig. 4b–e). The femoral head is medially directed with a small ventral flange at its medial end. The trochanteric crest seems to be well developed, but it is unclear whether the greater and anterior trochanters are completely co-ossified as in other parvicursorines such as *Mononykus*^2^, *Parvicursor*^6^, and *Xixianykus*^10^, or separated as in basal alvarezsaurs such as *Patagonykus*^14^, *Achillesaurus*^21^, and *Bonapartenykus*^18^. The trochanteric crest is separated from the femoral head by a shallow groove. The anterior trochanter is medially curved to make a distinct fossa which is located anterior to the femoral head. This fossa is also known in *Mononykus*^2^ and *Xixianykus*^10^. A sigmoidal ridge is developed on the anterior margin of the proximal part of the femur, descending from the thin anterior margin of the anterior trochanter to near the mid-shaft. The femoral shaft is anteriorly bowed to a lesser extent than most of the other alvarezsaurids such as *Mononykus*^2^, *Parvicursor*^6^, *Ceratonykus*^7^, *Qiupanykus*^20^, and *Xixianykus*^10^. An incipient fourth trochanter is developed on the posteromedial margin of the proximal third of the femoral shaft as a sharp ridge. A weak fourth trochanter is also present in *Mononykus*^2^, *Patagonykus*^14^, *Alvarezsaurus*^15^, *Shuvuuia*^4^, and *Xixianykus*^10^. On the other hand, there is no fourth trochanter in *Parvicursor*^6^ and *Linhenykus*^12^. The proximal part of the femoral shaft is subtriangular in cross-section, but the distal shaft is anteroposteriorly compressed being elliptical in cross-section. There is also a distinct ridge along the entire posterolateral margin of the femur, similar to that of *Xixianykus*^10^. It is most prominent around the mid-shaft and distally confluent with the ectocondylar tuber. On the distal end of the shaft, the femur has a wide popliteal fossa which is bordered by two longitudinal ridges. Between the two ridges, the lateral one is more prominent unlike in *Linhenykus* where the medial ridge is sharper^12^. It is not clear whether the popliteal fossa was distally open or not in life. Among alvarezsaurids in which distal region of the femur is known, *Parvicursor*^6^ and *Linhenykus*^11,12^ also have a completely open popliteal fossa. In contrast, *Mononykus* has a distally closed popliteal fossa^2^, and it is only partially open in *Xixianykus*^10^. The medial and lateral condyles of the distal femur are significantly worn off. The medial condyle is subtriangular in posterior view. The lateral condyle has a knob-like projection on the distal end comparable to, but relatively more reduced than that of other alvarezsaurids. It also produces a well-developed ectocondylar tuber posteriorly. Additionally, the lateral condyle has a small tubercle on its lateral surface, similar to the lateral protrusion in *Linhenykus*^12^.

The left tibiotarsus (Fig. 4f–j) is completely preserved. Its proximodistal length is approximately 1.3 times longer than that of the femur. The proximal surface of the tibiotarsus is oblique as the medial condyle projects proximally like in other parvicursorines^4,12^ except for *Xixianykus*, where the medial condyle is on the same level as the cnemial crest^10^. The cnemial crest is proximodistally short andanterolaterally deflected to a small extent. Medial to the cnemial crest is a nearly flat surface which forms the anterior margin of an accessory condyle (or medial cnemial crest). This condyle is also known in other parvicursorines^2,4,10^ but not in more basal alvarezsaurs such as *Patagonykus*^14^. The medial condyle has a convex proximal surface, but its posterior end is damaged. It is separated from the fibular condyle by a deep notch on the posterior surface. On the posterior surface, the base of the medial condyle is proximodistally elongated, extending more distally than the fibular condyle. The fibular condyle is rectangular in proximal view. The articulation surface for the fibula is mainly obscured, but it seems to be either flat or slightly convex. The cnemial crest and fibular condyle together define a wide depression between them. The fibular crest is not connected from the fibular condyle and dramatically reduced as in *Albertonykus*^13^, but not like the less reduced one in *Mononykus*^2^. It distally extends slightly beyond the level of the distal end of the fibula. The tibial shaft is straight along its entire length unlike the curved ones in *Parvicursor*^6^ or *Shuvuuia*^4^. The proximal part of the tibial shaft is subtriangular in cross-section, and it distally becomes more elliptical. A blunt ridge is developed along the anteromedial margin of the proximal third of the tibiotarsus. Distal to the mid-shaft, an additional shallow ridge is also present on the anterior surface of the tibial shaft. This ridge is medially located and becomes a sharp anteromedial margin of the distal end forming a thin flange. The distal-most region of the tibial shaft has a flat anterior surface and a convex posterior surface. Near the distal end of the tibiotarsus, a prominent tubercle is present on the posterolateral margin. The astragalus and calcaneum are completely co-ossified to form an astragalocalcaneum which is also partially co-ossified with the tibia as in many alvarezsaurids but unlike the totally fused condition in *Albinykus*^9^. The border between these elements, however, is mainly indistinct. The ascending process of the astragalocalcaneum is thin and laminar. There is a distinct notch at the medial margin near the base of the ascending process which occupies only the lateral half of the anterior surface of the distal tibiotarsus as in other parvicursorines^2,4,10,12^. A comparable morphology of the astragalocalcaneum is known in a Romanian specimen which was suggested to be from an alvarezsaurid^22^. The ascending process also arises from the medial margin of the medial distal condyle as in *Mononykus*^2^ and *Parvicursor*^6^, in contrast to the more lateral origin in *Shuvuuia*^4^ and *Linhenykus*^12^. In addition, there is a circular excavation at the centre of the base of the ascending process, similar to *Mononykus*^2^ and *Xixianykus*^10^. Like other parvicursorines, the medial distal condyle is more robust than the lateral one^4^. The medial distal condyle has its anterior peak on the medial end. It gradually descends laterally in contrast to the lateral distal condyle which is almost perpendicular to the anterior surface of the astragalocalcaneum. Between the medial and lateral distal condyles is a deep groove that extends to the posterior surface of the distal end of the tibiotarsus.

The proximodistally short left fibula (Fig. 4f, h–j) is nearly complete. It is generally similar to that of other parvicursorines in morphology being thin and anteroposteriorly expanded at the proximal end as well as having a distinct crest on the lateral surface of the distal shaft, which is for attachment of the M. iliofibularis^4,9,10^. However, the distal crest is not as robust as that of *Albinykus*^9^. There is a posterior projection at the proximal end of the fibula, but its posterior end is broken off and missing. The proximal surface of the fibula is concave having a depression in the middle. A sharp and proximodistally short ridge is developed on the anterior margin near the proximal end. On the opposite side to this ridge, the posterior margin also forms a short ridge which is not as sharp as the former. The fibular shaft becomes anteroposteriorly narrower towards the distal end where it tapers to produce a pointed end. The shaft is also straight unlike the posteriorly deflected fibula in *Xixianykus*^10^. The medial surface of the fibula is obscured by matrix and tibiotarsus, and the lateral surface is slightly convex.

The left tarsometatarsus (Fig. 4k–p) comprises of metatarsals II and IV, both of which are completely preserved. The distal tarsal is co-ossified with the proximal end of the metatarsus but does not completely cover it. Metatarsals II and IV are also partially co-ossified together at their proximal extremities. The proximal articular surface of the tarsometatarsus is subrectangular in proximal view. The distal tarsal cap borders medial and lateral fossae, both of which are triangular in shape. On the plantolateral corner, there is also a small depression which could be an articular facet for metatarsal V. A similar configuration of the proximal articular surface of the tarsometatarsus is also found in *Xixianykus*^10^. Despite its absence, tightly adjoined metatarsals II and IV indicate that metatarsal III was likely to be very short forming a highly modified arctometatarsal condition like that of other parvicursorines^4,7,10-12^. Metatarsal IV is only marginally longer than metatarsal II and approximately 3 mm shorter than the femur. However, with metatarsal III, the whole proximodistal length of the tarsometatarsus would have exceeded that of the femur as in other parvicursorines^4,10-13^. The proximal end of metatarsals II is medially deflected, but that of metatarsal IV is nearly straight. Both metatarsals have dorsal and plantar ridges which are proximodistally elongate. The dorsal ridges are prominent along the two-thirds of each shaft, whereas the plantar ones are developed only near the mid-length of each shaft. A small plantar flange is developed at the mid-shaft of metatarsal II. The dorsoplantar height of metatarsal IV is nearly consistent along the two-thirds of the shaft until it becomes distally lower. Metatarsals II and IV diverge to a small extent near the distal end. Unlike in other parvicursorines, the distal shafts of metatarsals II and IV are co-ossified at their plantar surfaces proximal to the divergence without any visible line of contact between them. The non-ginglymoid distal articular ends of metatarsals II and IV are similar to those of *Mononykus*^2^ or *Shuvuuia*^4^ in morphology. The trochleae of metatarsals II and IV are subtriangular in distal view. Metatarsal II has a robust lateral rim and a small medial rim at its distal end. Similarly, metatarsal IV has a larger lateral rim than the medial one although the difference in size is less conspicuous. The trochlea of metatarsal IV also has a small notch on its lateral surface forming a sharp flange which is absent in *Kol*^8^. There is no collateral ligament fossa on both trochleae of metatarsals II and IV, whilst in *Mononykus*, a lateral fossa is present on each trochlea of metatarsals II and IV^2^.

The preserved pedal phalanges (Fig. S2) are similar in morphology to those of other parvicursorines such as *Mononykus*^2^, *Parvicursor*^6^, or *Linhenykus*^12^. The degree to which collateral ligament fossae are developed varies from phalanx to phalanx as well as in the same phalanx. In most preserved pedal phalanges, distal hemicondyles are damaged and have plantar expansions that diverge medially and laterally, resulting in a triangular configuration in distal view. The two relatively large ones are possibly left pedal phalanges II-1 and II-2. The former is missing its proximal half, but the prominent extensor fossa is present on the dorsal surface near the distal end. There is also a distinct flexor fossa on the ventral side as in *Linhenykus*^12^. The collateral ligament fossa on the lateral hemicondyle is also well developed in contrast to the medial one which is nearly undeveloped. The following phalanx is consistent in mediolateral width and does not appear to have either an extensor or a flexor fossa. A prominent dorsal lip is present at the proximal end. Distally, the lateral collateral ligament fossa is deeply excavated. Phalanx III-1 has a mediolaterally expanded, trapezoidal proximal articular surface which is deeply concave. It is ventrally flat and missing its distal end. Phalanx IV-1 is short but stout. It has a broad proximal articular surface which bears a well-developed medial process and a less prominent lateral process as well as a distinct plantar excavation at the middle, all of which are also known in *Linhenykus*^12^ and possibly *Albertonykus*^13^. At the distal end, the lateral hemicondyle is broken off. The medial hemicondyle is intact and large but has a shallow collateral ligament fossa. The extensor fossa is very deep in phalanx IV-1. Phalanx IV-2 is also short and missing its proximal dorsal lip. Its extensor fossa is shallow and indistinct. The distal hemicondyles are asymmetrical, the medial one being much larger than the lateral one. The medial hemicondyle has a deeper collateral ligament fossa than the lateral one as well.

**3. Description of MPC-D 100/206**

MPC-D 100/206 is light to dark brown coloured (Figs 6 and S4) like the holotype specimen of *Nemegtonykus citus* (MPC-D 100/203). The morphology of MPC-D 100/206 is almost indistinguishable from that of *Mononykus* except for the distal end of the tibia which is completely separated from the proximal tarsals.

**Caudal vertebrae.** A total of seven partially articulated caudal vertebrae are preserved (Fig. S4). They are all procoelous and generally larger than the caudal vertebrae of *Nemegtonykus*. All of the caudal vertebrae lack pleurocoels. The most proximal ones are probably from the proximal or middle part of the tail. They are characterized by well-developed transverse processes which are close to prezygapophyses and inverse V-shaped ventral margins of centra that lack any furrows unlike in *Nemegtonykus*. There is no intact neural spine, but the dorsal surface of each vertebra has a anteroposteriorly long ridge. The postzygapophyses do not extend posteriorly beyond the level of the centra in any vertebrae. The more distal vertebrae still retain reduced transverse processes. However, the ventral margin of the centra is less angled, and a shallow furrow is developed on the ventral surface of each centrum. In these centra, the posterior articular surface protrudes to a significant degree. They are also proportionally more elongate in anteroposterior length and low in dorsoventral height than the more proximal ones. The zygapophyses of the distal caudals are short, and the neural spine is just a low ridge.

**Femur.** About a proximal half of the left femur is preserved (Fig. 6a). The femoral head and most of the greater trochanter are missing in this specimen. The thin anterior trochanter curves medially as in *Nemegtonykus* and other alvarezsaurids^2,10^. The femoral shaft is anteriorly bowed to a great extent, which is different from the slightly bowed femur of *Nemegtonykus* but very similar to those in *Mononykus*^2^ or *Qiupanykus*^20^. There is a week fourth trochanter on the posteromedial side of the proximal shaft. The cross-section of the broken end is suboval.

**Tibia.** The preserved left tibia missing its proximal region (Fig. 6b–d). The tibial shaft is straight like that of *Nemegtonykus* or *Mononykus* and has a subelliptical cross-section. The distal-most part of the fibular crest is observable on the anterolateral margin of the shaft. An inconspicuous ridge is present on the anteromedial surface of the shaft distal to the fibular crest. Unlike in *Nemegtonykus*, it is short and does not extend much both proximally and distally. At the distal end, both anterior and posterior surfaces are flat. The latter also lacks a distinct tubercle, which is different from *Nemegtonykus*. The distal surface is concave and sub-trapezoidal. Another notable character is the absence of co-ossification between the tibia and proximal tarsals. This is different from the partially co-ossified tibiotarsus in *Mononykus*^2^ or *Nemegtonykus*, but similar to the morphology of *Alvarezsaurus*^15^ or *Qiupanykus*^20^.

**Distal tarsal.** A small fragmentary bone (Fig. S4) which was associated with the left metatarsus (mainly with metatarsal IV) is here identified as a distal tarsal. It is thin, flat, and square in shape. It is also not co-ossified with the metatarsus unlike *Nemegtonykus*, but distal tarsals and metatarsals are not co-ossified in many alvarezsaurids such as *Mononykus*^2^, *Parvicursor*^6^, or *Qiupanykus*^20^.

**Metatarsus.** The left metatarsus is nearly complete (Fig. 6e–h) except for the proximal end of metatarsal II although metatarsals I and V are entirely missing. Metatarsals II and IV are closely attached to each other along the most of their lengths forming a longitudinal furrow between them on the dorsal and plantar sides. They are not co-ossified at any location, which is different from the partially co-ossified condition in *Nemegtonykus*. Proximally, metatarsal II has a medial deflection as in other alvarezsaurids^2,6,10,12^. Near the mid-shaft region, metatarsal II has a weak plantar flange. The trochlea of metatarsal II has a prominent lateral rim and a small medial one, which is common in alvarezsaurids. Metatarsal III is proximodistally short, not reaching the mid-shaft region of metatarsals II and IV. However, the exact location of the proximal end of metatarsal III is obscured due to its damaged proximal end. As in *Mononykus*, the shaft of metatarsal III does not overlap those of metatarsals II and IV. On each side of the distal articular end of metatarsal III, a deep collateral ligament fossa is present. The trochlea is not ginglymoid and has a pair of plantar ridges which nearly meet each other at the middle proximally. The medial surface of the distal end of metatarsal III is relatively enlarged as in other alvarezsaurids. Metatarsal IV has a semi-rectangular proximal articular surface. At the middle of this surface, there is a transverse ridge which could be a remnant of a distal tarsal. The proximal end of the shaft of metatarsal IV is significantly deflected in a lateral direction as in *Mononykus*, but unlike the nearly straight one in *Nemegtonykus*. The dorsoplantar height of the shaft becomes distally lower past the mid-shaft. Metatarsal IV extends slightly further distally than metatarsal II. The trochlea of metatarsal IV bears a small medial rim and a larger lateral rim, dorsal to which is a small flange.

**Pedal phalanges.** Left pedal phalanges IV-1 and IV-2 are mostly intact (Fig. S4). The former is slightly larger than the other one. The larger one has a deep extensor fossa, and its distal ends and collateral ligament fossae are asymmetrical. The smaller phalanx has well-developed dorsal and plantar lips at the proximal end.

**
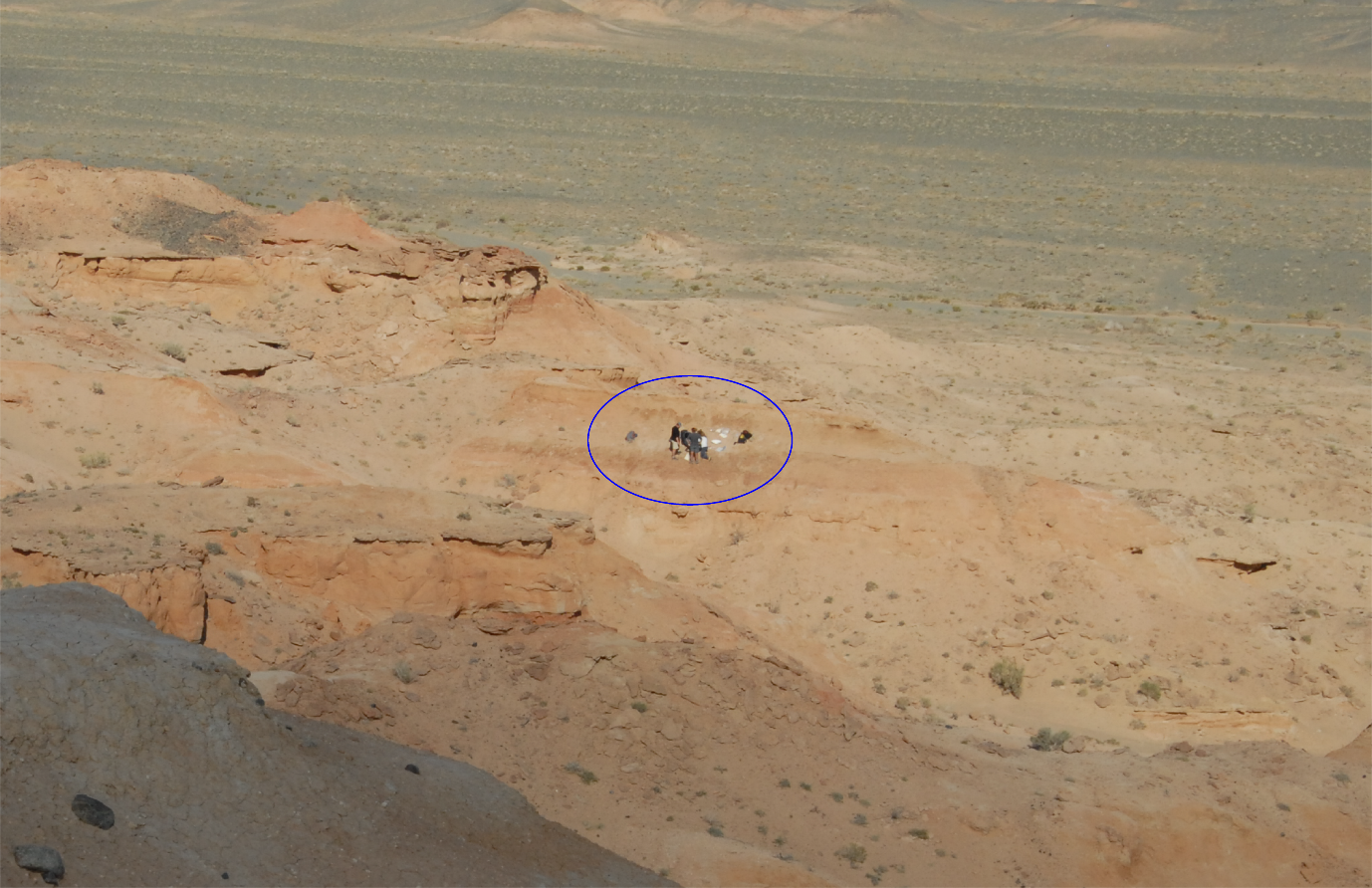
4. Supplementary Figures**

**Supplementary Figure S1.** Excavation site where the multi-species assemblage was found (marked with a blue ellipse).

**
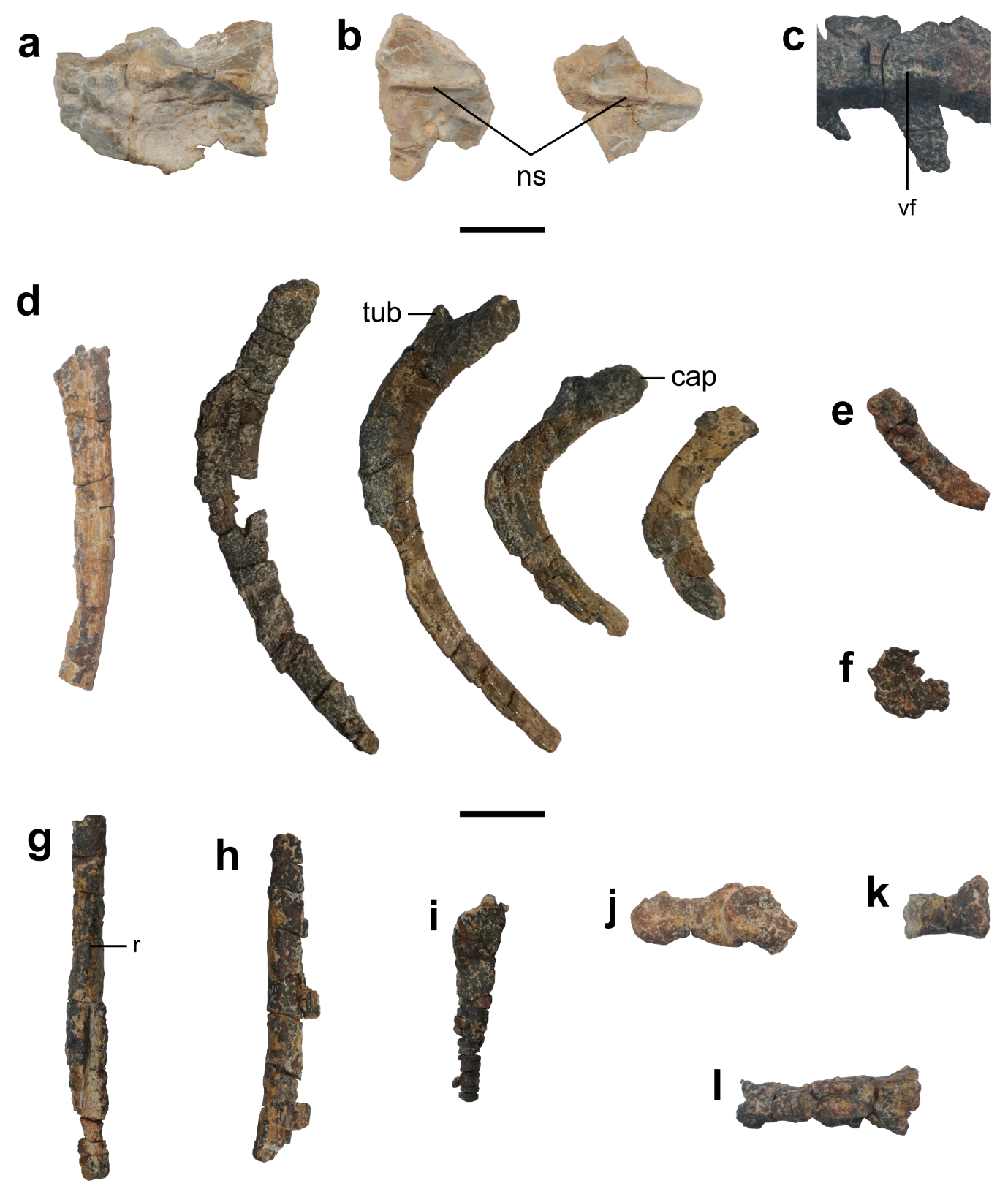
Supplementary Figure S2.** Additional elements of MPC-D 100/203. (**a**) ?fourth and ?third last dorsal vertebrae in left lateral view. (**b**) Two isolated dorsal vertebrae in dorsal view. (**c**) Caudal C in ventral view. (**d**) Isolated dorsal ribs. (**e**) Proximal chevron in left lateral view. (**f**) Mid-caudal(?) chevron in left lateral view. (**g**) ?left middle pubic shaft in lateral view. (**h**) ?left distal pubic shaft in lateral view. (**i**) ?right proximal pubic shaft in medial view. (**j**) ?left pedal phalanges II-1 and II-2 in lateral view. (**k**) Left pedal phalanx III-1 in lateral view. (**l**) Left pedal phalanges IV-1 and IV-2 in dorsal view. Abbreviations: cap, capitulum; ns, neural spine(s); r, ridge; tub, tuberculum; vf, ventral furrow. Scale bars equal 1 cm.

**
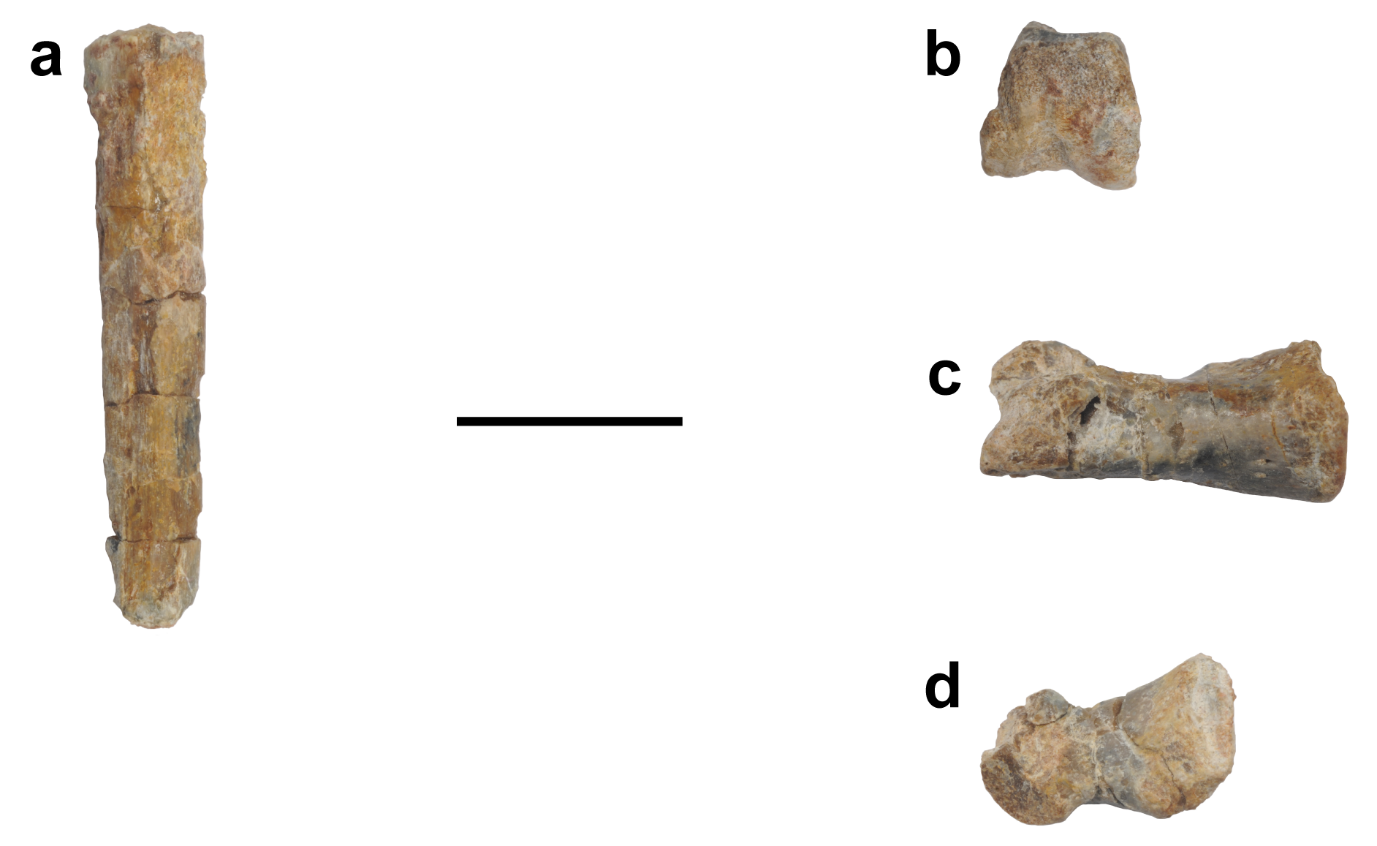
**

**Supplementary Figure S3.** Additional elements of MPC-D 100/207. (**a**) ?left pubic shaft in posterior view. (**b**) Right metatarsal III in dorsal view. (**c**) ?right pedal phalanx II-1 in dorsal view. (**d**) ?right pedal phalanx IV-1 in medial view. Scale bar equals 1 cm.

**Supplementary Figure S4.** Additional elements of MPC-D 100/206. (**a, b**) Proximal caudal vertebrae in left lateral (a) and ventral (b) views. (**c**) Two mid-caudal vertebrae in left lateral view. (**d**) Isolated mid-caudal vertebra in left lateral view. (**e**) Left distal tarsal in proximal view. (**f**) Left pedal phalanx IV-1 in lateral view. (**g**) Left pedal phalanx IV-2 in lateral view. Abbreviation: tp, transverse process. Scale bar equals 1 cm.
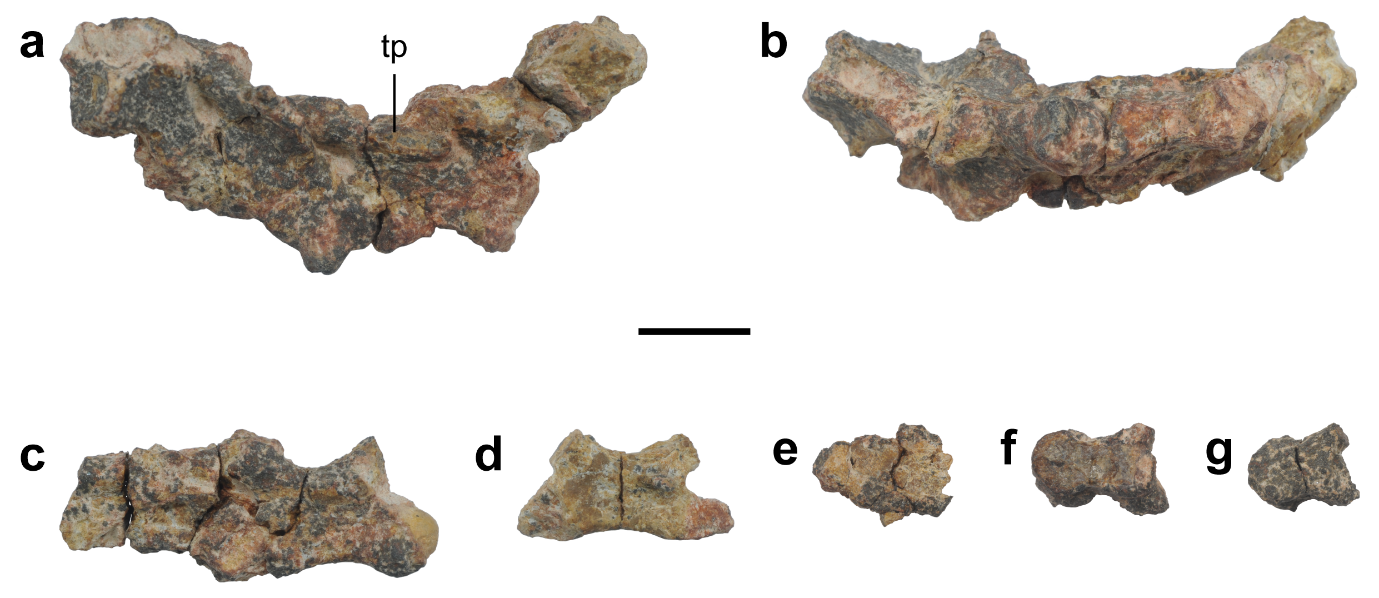


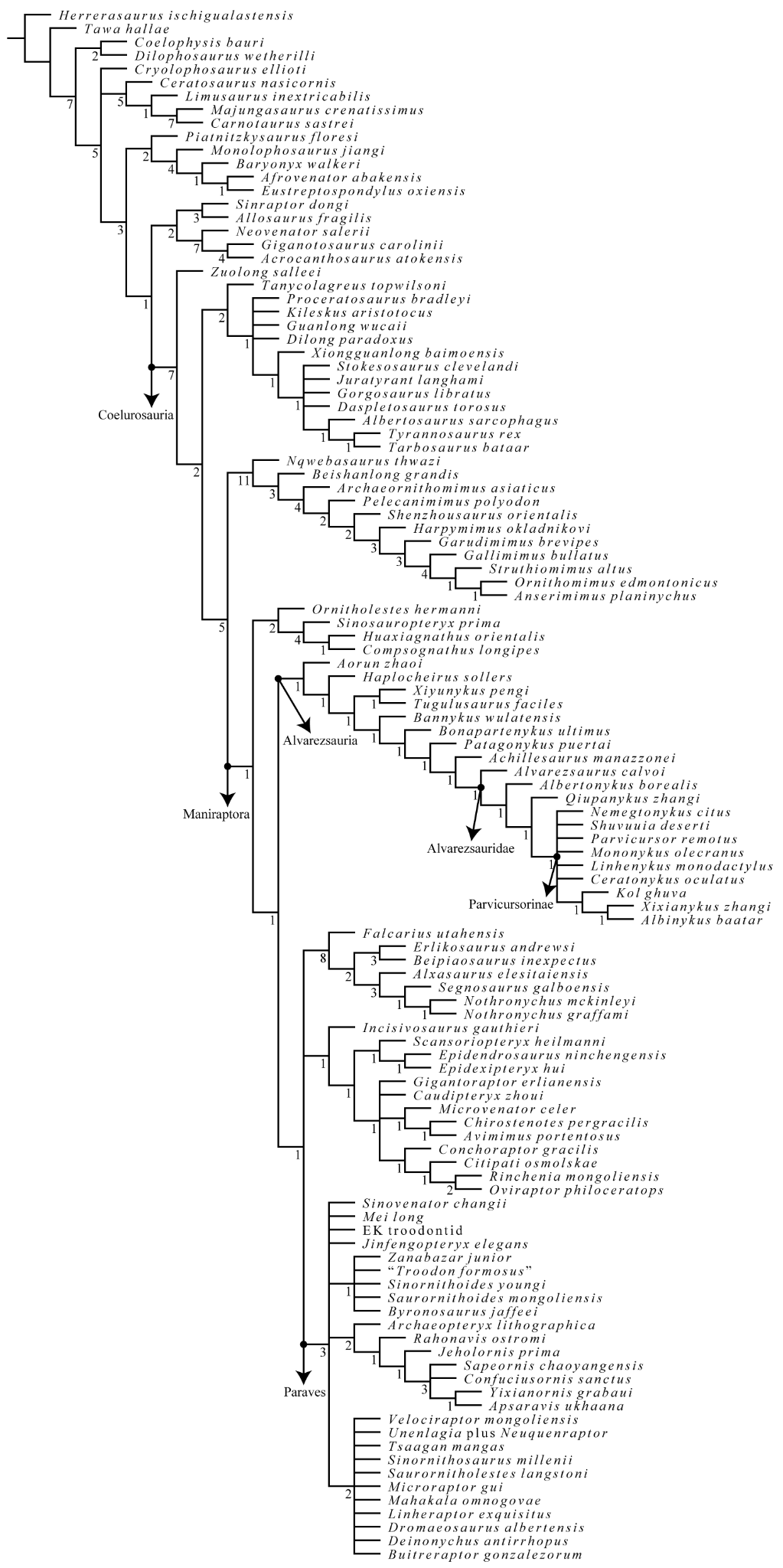


**Supplementary Figure S5.** Strict consensus of 500 most parsimonious trees with 3226 steps (consistency index: 0.217, retention index: 0.604). Numbers at each node indicate Bremer support values.


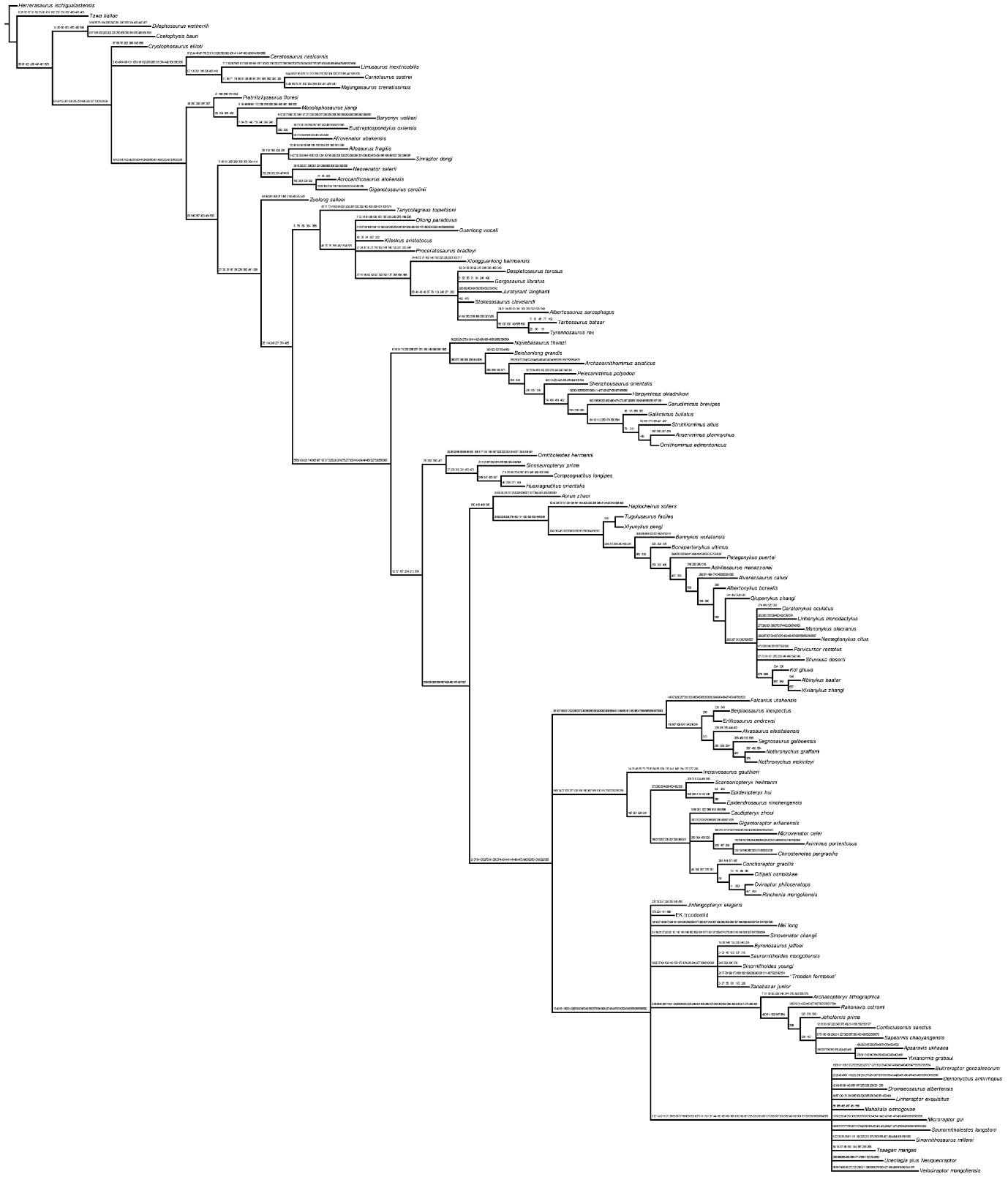
**Supplementary Figure S6.** Strict consensus tree with common synapomorphies.

**5. Supplementary Tables**

| **Element** | **Length** | **Width (mediolateral)** | **Height (dorsoventral)** | **Circumference (mid-shaft)** |
| --- | --- | --- | --- | --- |
| ?Third last dorsal centrum | 18.17 | 6.54 (anterior end), 8.1 (posterior end) | 9.98 (anterior end) | - |
| Penultimate dorsal centrum | 14.8 | 6.24 (anterior end), 8.22 (posterior end) | 9.55 (anterior end) | - |
| Last dorsal centrum | 17.35 | 7.83 (anterior end), 11.45 (posterior end) | 9.45 (anterior end) | - |
| First sacral centrum | 14 | 10.73 (anterior end) | 8.81 (anterior end) | - |
| Second sacral centrum | 14.27 | - | 8.66 (anterior end) | - |
| Caudal A centrum | 16.84 | 7.34 (anterior end), 6.29 (posterior end) | 9.08 (anterior end) | - |
| Caudal B centrum | 15.12 | 7.15 (anterior end), 6.26 (posterior end) | 7.87 (anterior end) | - |
| Caudal C centrum | 15.37 | 6.27 (anterior end), 5.99 (posterior end) | 7.9 (anterior end) | - |
| Caudal D centrum | 14.78 | 5.95 (anterior end), 5.38 (posterior end) | 7.8 (anterior end) | - |
| Caudal E centrum | 13.97 | 5.7 (anterior end), 5.68 (posterior end) | 7.9 (anterior end) | - |
| Caudal F centrum | 14.14 | 5.93 (anterior end), 5.93 (posterior end) | 7.62 (anterior end) | - |
| Caudal G centrum | 14.24 | 5.94 (anterior end), 5.98 (posterior end) | 6.37 (anterior end) | - |
| Caudal H centrum | 16.02 (distorted) | 5.93 (anterior end), 6.03 (posterior end) | 6.21 (anterior end) | - |
| Caudal I centrum | 15.68 | 6.07 (anterior end), 5.98 (posterior end) | 5.58 (anterior end) | - |
| Caudal J centrum | 15.86 | 6.31 (anterior end), 6.21 (posterior end) | 5.75 (anterior end) | - |
| Caudal K centrum | 16.2 | 6.53 (anterior end), 6.04 (posterior end) | 5.78 (anterior end) | - |
| Caudal L centrum | 16.24 | 6.55 (anterior end), 6.33 (posterior end) | 5.63 (anterior end) | - |
| Caudal M centrum | 16.66 | 6.01 (anterior end), 6.09 (posterior end) | 5.4 (anterior end) | - |
| Caudal N centrum | 14.31 | 5.61 (anterior end), 5.45 (posterior end) | 5.16 (anterior end) | - |
| Caudal O centrum | 15.69 | 6.56 (anterior end), 5.77 (posterior end) | 5.64 (anterior end) | - |
| Caudal P centrum | 15.27 | 5.84 (anterior end), 4.78 (posterior end) | 5.43 (anterior end) | - |
| Caudal Q centrum | 16.23 | 5.02 (anterior end), 4.91 (posterior end) | 5.02 (anterior end) | - |
| Caudal R centrum | 15.54 | 5.41 (anterior end), 4.86 (posterior end) | 4.97 (anterior end) | - |
| Caudal S centrum | 13.55 | 5.19 (anterior end) | 4.85 (anterior end) | - |
| Caudal T centrum | 12.58 | 4.92 (anterior end), 4.95 (posterior end) | 4.8 (anterior end) | - |
| Caudal U centrum | - | 4.79 (anterior end) | 4.31 (anterior end) | - |
| Acetabulum (left) | 16.92 | - | - | - |
| Femur (left) | 116.43 | 20.57 (distal end) | 9.69 (anteroposterior diameter, mid-shaft) | 34 |
| Tibiotarsus (left) | 152.72 | 16.55 (distal end) | 8.64 (anteroposterior diameter, mid-shaft) | 30 |
| Fibula (left) | 38.91 | - | - | - |
| Metatarsal II (left) | 112.94 | 9.11 (proximal end), 8.17 (distal end) | 9.55 (proximal end), 7.52 (distal end) | - |
| Metatarsal IV (left) | 113.59 | 8.61 (proximal end), 6.9 (distal end) | 9.5 (proximal end), 7.14 (distal end) | - |
| ?Pedal phalanx II-1 (left) | - | 6.78 (proximal end) | 7.57 (proximal end) | - |
| ?Pedal phalanx II-2 (left) | 14.2 | 6.35 (proximal end), 5.65 (distal end) | 6.14 (proximal end, damaged), 7.69 (distal end) | - |
| Pedal phalanx III-1 (left) | - | 8.97 (distal end) | 7.46 (distal end) | - |
| Pedal phalanx IV-1 (left) | 12.5 | 8.16 (proximal end), 5.83 (distal end) | 8.05 (proximal end), 6.37 (distal end) | - |
| Pedal phalanx IV-2 (left) | 11.39 | 4.61 (proximal end, damaged), 5.95 (distal end) | 6.73 (proximal end), 6.18 (distal end) | - |

**Supplementary Table S1.** Selected measurements of MPC-D 100/203 (in mm).

| **Element** | **Length** | **Width (mediolateral)** | **Height (dorsoventral)** | **Circumference** |
| --- | --- | --- | --- | --- |
| Femur (right) | 115.16 | 18.01 (distal end) | 9.14 (anteroposterior diameter, mid-shaft) | 29 |
| Tibia (right) | - | - | 7.69 (anteroposterior diameter, estimated mid-shaft region) | 30 (estimated mid-shaft region) |
| Fibula (right) | 37.14 | - | - | - |
| Metatarsal II (right) | - | 9.64 (proximal end), 7.38 (distal end) | 11.16 (proximal end), 7.75 (distal end) | - |
| Metatarsal III (right) | - | 7.01 (distal end) | 6.27 (distal end) | - |
| ?Pedal phalanx II-1 (right) | 16.4 | 7.13 (proximal end), 6.22 (distal end) | 7.42 (proximal end), 5.37 (distal end) | - |
| ?Pedal phalanx IV-1 (right) | 11.63 (damaged) | - | - | - |

**Supplementary Table S2.** Selected measurements of MPC-D 100/207 (in mm).

**6. Character modification statement**

Below is the character from Xu et al.^23^, which is modified in this study.

468. Shape of pubic peduncle of ilium

0 transversely broad and roughly triangular in outline

1 anteroposteriorly elongated and narrow

2 greatly reduced into a knob-like structure (newly added)

Justification: state 2 is added in order to incorporate the greatly reduced small pubic peduncles in *Qiupanykus*^20^ and *Nemegtonykus*, which are neither broad or elongated.

**7. Data matrix**

Herrerasaurus_ischigualastensis ??000000?00??100----???0010-00000000000????0?00000001?2010000000000-0--01100--00100000??-?00?0-00?00??00?1????01???11??000?0?0000?0?????0???000????01?1?0?0?0-000????0???????????0???1?000-0?0011???????010??000??????????1010?000?010000000???0?000??00-0000100?0????0010?001000?000000000000000000000100?0?01?1??0?000?00???200000000?000?0000?????????00??????????0??0-???00?1000010?001110?10000100??0100?0?00001?0??01000-0?0120000000110000?11100000???0000?0-0??0????00-000001001????00??01010???00000?0101????00-1?1?0-??010000?2000000000000000?0000----0?0??100????0010000000?000?0-0?00?0000?000?01000-

Dilophosaurus_wetherilli ??001020?00??0?0----1?????0-010?0010011{01}0{01}1??0?0?1001?2010001000010?0--1111???0010?0?0???00010-01?0????111????000?011????????0000?0???0?1???000???1?1?0?0?0?10000??101?????0???????1?1?1?0-???0???0-10?001010?01??????????1010?000?1000000000??1?10???00-0110110?1101?0011?0011?0??0?000110000200???10?10000????1100?030?00???0?0000020?000?01000?0???10?0???????????0??2000000100???00?0??100?100001000?0100?0??010?01?00?000-1?0020100000110000?1?11000?????11000-???0????1?00?0001101????100?00-00???100?00?100????10-????0-??11000001000000000?0000??00110?000?0??100????001000000??0?0?10??0??0000?0?2001000-

Coelophysis_bauri ??000011??0??000---????0?00-0101001?1?10???0?00001001????0000000000-0--11100--00100??0??-000?0-01?00??0??0?????0???????0?????0000?0???001?????0?????1???0?0?1000?????????????????0?001?000-0?0011???10?100-??000??????????1010?010?020000000?????000??00-1??0100?11000?02??0010?0?100??00{01}00002001??000????0????00?0?03??00???100000020?000100?01?0??????00??????????0???????001100?000?0?01?0?10000100??0100????000??1?000000-1?0000?000?0?1000??111100?0???001000-0000????100000001101???000?000-00???0001101100????10-1?0?0-??1100?0??0?0000000?00101?0?110???0??0?001????001000000??0???10???0?0?0????1001000-

Tawa_hallae ??0001201000??00----01001?11000?00000?1????0?000?0011?200?001000010-0--12100--?1100??0???000?0-00?0?0-??11010000??0????????????0?020?00?1???000???????000??1??0??????1?????????????10??000-00000-00-100?00-??0????0????????00??00002000?0000?0000?000100-0???????1100?00??00?1?????0???0?????0?????????1????1?????0????????0???????0??00????0?0?00????????????????????????????1?10000100?00100110000?????????????0??1????000???10??2?0000?011100001110?000000000000-0???0--?0?00??00000100?000?0?110????????10?????????????????????001??2000000??100????????????????????????0001000000000?010-?00?00???00?1???????

Carnotaurus_sastrei ??10000000001000---0??00?010000100010001???1?1001110??200000000?000-10101000--?10-1100000001?1101100101000010000??00-???0????1000??2??0??????0?00????00?1001??0?0????????????????1?101100??00?01000-100?0100-0??0?11101000101000??01000000?0?0000000???0-000010101101?10?00??11102?1011100?000100?0010?1000010011?110040??11?0??????00????0?????0000???0?????0???00--0000-000?01000011010?0221?0110?2000000-000111000----????????1?0???01?????????????????????11000-0000??????00?00??00??0?000000100????1000000100?000?0-00000-??000?10000?0111011??011?00?110?0??????????????????????????????????????????????????

Ceratosaurus_nasicornis ??10100010001100---00?00?0100000001000010001111010101101-1000000001110?11100--0010010000000010-011101010010100010?00-0?00?0??1000?02??0?1???000???0???1?1???10?00???0????????????1?????000-???0???0-10?00100-0?0??????100?100??000?11000?0000??0?0?0??00-0000111?11011000000011??0?0011101?000100?00???10000000110110040?01?0?1?000?01000?0?01000010??10000??????????00020000?0100?0?1000?????????????????10001100101????0100??10000010001011100??1??????2????1100??0000????1?00000010010000000000-000101000100100000010-00000-0?11000000000011011000111011110?0?0000?1000???001000011?????????????00?0???????0?0?

Limusaurus_inextricabilis ??1000101001110101?00?000?11?0?1?01?000?---00000?000??01-?000000000-10012100--00120??0???00000-00?1????0000?0?020?00-?????01?000?????????????????????????????????????????????????0?20-?00??01?1110-?0-1000-??0?10?0??0????11------1-----------------------000??0?1101?1?2?0000-??01?0???0??????????????????00???1????0??????????10000?000???020000000-0??1101000??0-10002000000110000100001?10011011?000?00000?000000----?0000--010001000101{123}100-00011--02???000000-000110001000?1001001001000000100???0?0000?00-??000?0?00?00?1???00?010?0?0?????00011????????????0??10??????1???????001?0???00??0??00???2?00000-

Majungasaurus_crenatissimus ??10100010001000--??100000100001000100011100?1011010112010000000010-101?1010--010-1100000011?11011111011000100010?00-0000001?1000012??001?00000001??1?????0???0?0?????0-?????????0?10110???0?00?????10?0?100-0100001001000101000000110000000000000000100-0000111010-1?10000?011?02000111010000100100101?0000001010110030???1?001100002000000110000000?100100??????????000-00000???000?0?00022100100120000??????????0?????????????1?0???01???11????????????????010010000010001000?0001001000000100????????????0???0??????-??0???????????000000??00100011101101010000111100010000100010??0??0???0000000?0??0?0000011

Cryolophosaurus_ellioti ???????????????????????????????1??1?1??000??0??0????0?2?????0?00001011??1100--001000?????00000-01?00??10?10??00????10?0?0???????0???????????????0???10101?????000??????????0-????0???????????????????????0-0-110??0???????????????0{12}??0?0000?????????????????????10-1{01}????00?????0?????0?????0?00?00???10010??0?1?10?0????????00???0????????00?0?0????0?00??????????????????????????????????????0?????????10000?????????????????????????????????????????????????00?????????????0?0??1??10?????000????????00????????00????10??????01001?010??011?0??0000??????????0??????????001100000?????????????????????????????

Monolophosaurus_jiangi ??001000100101?10000?00?01110001000?0000??001111000100201100000001111??11100--0010000000-000110011000-0011000001??010????????0000?????0?1???00??????1010100?100000-??????????????0?0011110-?0000-00-100?0100-1????0100211?1010000002000?00000000000?010100??1101?10-1?100?01011?00010?100101001000000011?000000?10010030?00?0?????0?02????0?????0?????????????????????????????????????????????????????????????????????????????????????????????????????????????0100100000100100?0?01000010000000000-??0101000100100?00110-100?0-???????????????????????????????????????????????????????????????????????????????????

Piatnitzkysaurus_floresi ?????0?????????1??????????1000????110?00100?1??????????????????????????????????????0???????????????0????????????????????????????0?????0?1???0?????10??1?1?0?10?0010?010-?????????00????0?10????????????0??????????????????????????0?1?01?00?0??0?0????0100111110?10-1010?1?0011????10??001?10010???????11000????1?00??30??0???0???0?02????0??????????????????????????0??21000?0?0????00?0??1?0?10?00???0??100????0????????????????????????????????????????????0100??0????????????010000??????????1?00????0000?010?????11-1???0-??110010?0100010?0100??0??10111???0?0????0??????????????????????????01?0???????????

Baryonyx_walkeri ??0101201001110??????10?0?11?0?000??00100000101????0??2??????0??0?101??10100--0010011???10?000??1????11???????020?010??????????0000000??1?10010???0?10101000110000-0?10-00-10??0?????11110-?0100-00-10010??1111000???0101?103000000?20000010102101000100-1101111?10-1010?001011?00010??01121001100??000110000??0100100?????????0??0002???????????00???01??0?101010???0000-???00?00?0?10000?2010?02010001012100110101??????????????????????????0000????0100?????1?0??????????1000???0000100????00?0-????0????0?0100000?100?0000-0?010110??????1000100??????????????0?0100000?00????????????????????????????????????

Eustreptospondylus_oxiensis ??0??020100?1100----00000?1100??0001001000?0???????1??????????000?1-11011000--0011011?00000010-010?0????010?00000?00-????????0??0?????0?1?00010??01010001?0111?100-??10-00-10000??0??1?110-?0????0101001?????????????0?????010000002000?0000000101000100-0110111010-11100101011?00?10??00100002000??000110000??01000?030?00?0?0?{01}00002???00?00????????????????????????????????1?00?0?100?0?200010001?0000??????????????????????????????????????????????????????10???0??0???01000?000000110000000010000001010000100010?0?01?000-010100100000001000100010000011100000001100000000100011??00??????00000100000?001000-

Afrovenator_abakensis ?????0???????1?101001??0??11010100110?00000?1???????0?201100??0?001010011100--00?????????00010-01??0????010000001?010????????0?0??????????????????????????????????????????????????????????????????????????????????????????????????02000100000???????0100-0110110?10-1?0001?1?11???010??0?{01}?10?10???????1?00???????0????????????0???0??0?0?0?100???????11?00??????????????????????????????????0??0?01???????00?????????1???011??1??00???????121000?2???010000000100100??0????00?00010000100?0000000-0?0?01???000100010011010000-?0?0?1100010001000100????0?0??????001????00?0000100?111??1?0?110????01?????2???0???

Allosaurus_fragilis ??0010{01}01001010?0000?0000?1000010?110?0100100001?1?011?1-000000000101???{01}100--?111010000-01000-010?0???01?0100010101120?00010??0001001001?00?000010?00101001100100-1?10-?0-0-?000000010000-?0000-00-10000110-11?000100101010200000021001000000000000010100000111010-1000000101100001001011010000100000010000001010010030?00000000000020000001001000???1?0100??????1000002100001010000000000100000000100000000000000010100001110100000100010121000021110000000001001010001000100000100001000000000101000?1010000100000011010000-00010110001100110010001000101110000?00010000000110001110?1?00110000001010?02001000-

Neovenator_salerii ??00?01010000101000010000?1100010011000101000101110????????????????????????????????????????????????0???????????????????????1?????????????????????????????????????????????????????????1?100-?000??00-??00??????????????????102100000?100100000000010?010100001111011111100001011?00010??00200000010??001101101??1100100?0?10???????000100000100010?10??100100?????????0002100101?10?0?1010?????????????????????????????????????????????????????????????????????0110100??010001000??000001000??110010100101010000100000011000000-00010120001100110010101010111110010001101001????100?????01??????000?01?10002001000-

Sinraptor_dongi ??001000000000?10000000?010-000110110000?0000001000000200000000100101??11210--?110011000-010010011000-00010000010?011300000100001000010?1?00000??10?00101000100100-??10-?0-0-?0?00?1011000-?0000-010100?01110010000??010101010000002100?00000000000?010100110111010-10100001011?001100101101001010001001100000101?010030?0000?????0002?00?0??1?1001???1??1??1?0000???0????????1010?00100?0???????0??????????????????????????????0????11001012100?????1000000100100101001???000?0??1000010000000001100??11010000100000011010000-00010?1000110011001000101010111?000000?00?0000001000011000?00110000001010?0100?000-

Acrocanthosaurus_atokensis ???010100?0??101?0??10?0?10-?0?1?0110001001000?0?0?0112011??0?00001010??1110--?11?0??0???01101111?00??10110??01001?1120?0??1?0??0?????0?1?0?0?????1??0101?0?10000????10-?????????0?????200-?0000-?10???0?101011???0??0?0??101??000021001?00?0?0??1?0???1000?1??1?111110??001?11??0?10????2?1???01??????10?1?0??0??01?0?1??????????0?02??????10?1?0????1?010??????????0???1000?10100?010?0?0100000??10????02??00?101??0?0??011101?000?10001002200002??100?0???00???10??????????????10??????????10?1010??0?0{01}0??0????00?11?10?00???01012?1001?011?0??00100?101?????0?0?????0??0??100????001?000-00??0?1010??2?01000-

Giganotosaurus_carolinii ???0?00????????0????10????10???1?001000100??01?1?0????2???????????1010??1110--??1?0??????01101111?01??1???????1?0??10{12}??????????1????????????0????0???101????????????1?????0-????1?????201-?0?0???10???0???10?11????????????1???000200?0?10?0?0??0?0???110001??1?111110??{01}?1?11??0?10??0?2?0???0???????10010????1?01??????0???0?000?01??????000???0???1000???????????0??????????10??010?0?????????????????????????????????????????????????????????????????????0???10000?110?1??0?0?000?11?????10?1010??010100?0???????11?1???????01012?100??01??0?????0??10111???0?0??1?00????1???????????????????????????????????

Aorun_zhaoi ??00??00?10?110101001100??0-00010011100????00000?0000?2000100000000-0--1110110?1100??0???100?0-00??????010??0?0000?11????????0?0?????????????????????????????????????????????????0?10??010-00000-00-0-0?01?0-1??000??111?01010001002001-0010001100000100-0???????1101?101001?11010110000?00?00?00000000??0?000??00????????????????0010???????????????????????????????????????????????????????????????????1??0??00???1?10000110-10110111?0?00221001212101000110?????????????????????????????????????11???????????????????????????????????????????????00000???1000020?0??????000?100110?00100011?00000?01000?001000-

Ornitholestes_hermanni ??00?0000?01?00101011100??10000?01101000000000?0?001110001100000010-10011100--00101?0100?10000-000201000?001000000011{23}0????100000???0?0?????0?01001111101?100-????-????????????????0010?10-?0000-00-100?00-0-11100011010001012011102101-001000000?010100-????????10-1?101200011??111100?11?1001??0001001??100001010110?0???10?0?10?0020000?10?0??????????1?1?????????????????????????????0?10?11000010001?0??0?0?0?0?????0??????????????0??????????????????????1?0101??0100000?0?0010011000000?10????010?01000010?000001?10000-0????????????10?0?10?????????????????00??0?????????????001??????0?100?0?0?0?0010?0-

Zuolong_salleei ??00??00?00?010101001?0?0?0-0000001110000000??????00???????0??010?0-0--1?10111011????00??00000-0?1100-00?100000001011300??0????0??????????????????????????????????????????????????????????????????????????????????????????10100001020?0000100???????010100???????10-0?101100011??0?100???00?001???????????????????????40?001?000??000000??0?00????????????????????????????????1?10???1???0?10001???0100???????00?000?????????????????????????????????????0???00100??????????00?0???0???100?00000011?0010112?00???????????????????1?011000100001001000001010111?0???011??000????????????010????100010101000????0???

Albertosaurus_sarcophagus ??1????0000011??0????10?1???0??0??0100000101110?11??002011001100011111011201??000-12000010111100112010101?00101010010300000100000000000?1?00010?01??1000?00110000100?10-?00???0000?001??00-?0?00-00-10000101011?011100???010??00?10?00??0?0000000?0001010???011?0???????0000011?00??????0?0?00?????????1??000??0?00?0041?0?00???10??0??00??0??0?00?0????0??0000001??000020000???00?0??0000?11?001????????00??0?00??01000000010-?0???????????3200?0??-?0000????????10101?100000000?20???1000000?0010??0001???00010000000102000101?1101100110000100100000001?1110011001?1000100?2000110?001?0011000001111000?0?1000-

Daspletosaurus_torosus ??100020100111110100??00?10-00000101?000???11101??????2??0001100011111011101100011?01001?000?0-011201010110?1?101001030???01?0000????????????0?00????????????????100?10-?0-10?0000???1?000-0000??00-1000010101????????211?10100001020000000000000000010100??????????????0???0????0???????????????????????????????????0????????????????????????????????1?111?????????????2??????????????????????01??????????????????????????????????????????????0????-????????0????????????????????20???????????????????????????????????????????????01?0002???1?0?10?????01?111???2??????????????????????1????????????1????????0?0-

Dilong_paradoxus 0-100020000110?101001100?10-00000011100????010210100012011001100000-11011100--0010001100000000-001200-0001?00010??011??0?????0000010010?1?0000000?????1?1??1??0001??0????????????0?0-11000-00000-00-0-0000-101111111101010101000010200000000000100000100-0???????10-1?100000010??0010???0?01001?????????????????1????0??????????????0?0??????2?000?0??1??????????????0????????1?10?0000?01?1???10001????????????????????????1??1??10?110010022?0??21????00000??1?0??1??010000??0??20000100?0?0???11??????????????0??????0??0????11?01?0001?00??0?10??0?001?111??010???10???0002000111??01???????0?00?0??0?2???000-

Gorgosaurus_libratus ??101020100011?101001100110-000000010000???1110?11101?001100110001111?11110????0??1210000001?0-00?20101011??10?0?????3?0000100000000??0?1?0000000?????????????000????????????????0?00??000-00000?00-1000010101110?0??0????10100001020000000000000000000?????????0???????0?0??????00??110??0??0000000?0?????0000?1????0???????????????????????????000??????????????10??????????1??????0?????????????????????????????????????????????????02???32?????????????????100101011100????0?12100?100?000?00101?0001???00?10010?0010????????????????????????????00???????????0?0?10???????????????01000110??????1????????0?0-

Guanlong_wucaii ??10100000011111010011001111000000111000010010110001012011000100000-11011100--0010001000-00000-0012010001101101200011300?10??000000000011000000001101000100011000100010-1001000000?001?000-00000-010100?00-10111??1??0????101000010200{01}1001000?000000100-1??????010-10101000010?1001000010010010000000010010001110010030?0010?00100000000?0??2000000?????11??????????00020000?1?10000000?0110011000010001110001000001010000010-10010011001002100002121000????00100101010100000000021001100100001010110001000000100000000-100010011?01100011001100100000101111100?2000?1?00100011001111001?00110000001010002101000-

Kileskus_aristotocus ??10??00?00?0?11000011000?0-00??011110000100??????01????????????????????????????????????????????????????????????????????????????????????????????????????????????????????????????????????????????????????01?10?01???????????011000102100000000???????010??0????????????????????????????????????????????????????????????????????????????????????????????????????????????????????????????????????????????????????????????????????????000?????????????????????????????????????????????????????????????????????????????????????????????????????????????????????????????????????????????????????00??????001?1???????????

Proceratosaurus_bradleyi ??10??000000011101001100111000?001110000???0??1???010?001100010000?????01?0????01???????????00-?0??????0110?0000???112?0??0??0?00011010?1??011????????????????0??????????????????0?001?000-00001000-100000-10111100??0???01010000102101-0010000000010100-0????????????????????????????????????????????????????????????????????????????????????????????????????????????????????????????????????????????????????????????????????????????????????????????????????????????????????????????????????????????????????????????????????????????????????????????????????????????????????????????????????????????????????????

Juratyrant_langhami ?????????????????????????????????????????????????????????????????????????????????????????????????????????????????????????????????????????????????????????????????????????????????????????????????????????????????????????????????????????????????????????????????10-1?100?00011?00???0?0010000100000??1?0???0010?00??030?001000???000????00???????0???1???????????????????????????????????????????????????????????????????????????????????????????????????????0100110??1100?0100??21001100?00000011?1000?00001010001000001?0?101????????0????11001010101010110001??0??????????????????????????????????????????????

Tanycolagreus_topwilsoni ??1??000?00?00????????????????????????????1?0000???0???????0??00000-101122011000100??0???00?11001?????????????0000010?????????????????????????????????????????????????????????????????????????????????????????1000????101??01??0?10??0??000?0????????????011???1??0-??1????????????????0?0?000100??????100110??0?001?0?0??????????00?0???????20?00????1??????????????000200000111000000000110111000110001110000000101?11000010-100000110000022100020?100000000???????????????????????????????????11110101???00??????????????????1010110001100010010000010101110001000110001000200011110010001000000010?0002001000-

Tarbosaurus_bataar ??0???00000011010000?100110-000000??0000????110010100120110011000?111101???100001??01000-0?1010?112?111011001010??01031000010000?000??0?1?10010001?000001001??0000-??????0-?????0??0011?00-?0000-00-100001?101110111001010101000010200??0?0000000?00010??????????????????????????????????????0???????????????????????0?????????????????????????????????????????????????????????0????0??????????0?????????????????????????????0-??1?0???02???32?0???????????????????????????????????????????????????????????????????????????????????????????????????????????????????????????????????????01?????1??????1??????000?0-

Tyrannosaurus_rex ??10?0000000110101011100110-00000001000001011101101000201100110001111101110110?111101000-011?1001120111011001010100103?000010000001001001?10?0000100000010010-0?010?010-?0-100000000011000-00000-00-10000101011?011100101010100001020000000000000000010100000111010-11?00000011?0001011001000000000000010000000010010031?00000?0100000100000020000000-100110??????10100020000010100000000001100010012??00010001001001?0?000010-101000------032000021--00000?00010010101110000000?12000110000001001011000101000010000000102100101011012001210010001000100010111?0010?0??0?0100020001101001?0011100001-110002000000-

Stokesosaurus_clevelandi ??????????????????????????????????????????????????????????????????????????????????????????????????????????????????????????????????????????????????????????????????????????????????????????????????????????????????????????????????????????????????????????????????????????????????????????????????????????????????????????????????????????????????????????????????????????????????????????????????????????????????????????????????????????????????????????????010010101110010100??21001100100010??????????????????????????????????????????????????????????????????????????????????????????????????????????????????

Xiongguanlong_baimoensis ??10?000??0?1?1?0????100110-?0??011?000001?0102001??0?0011001100010-1?1??100--?0111??0?00001???0?120111011??1000000103?001?1?0000000???1100000???0??10001000110000-???????????????????????????????????????????????????????101??01102100000000???????0100-0000111010-1?10000001000001001000000010?100100100110?10100100????????????????????????????????????????????????????????????????????????????????????????????????????????????????????????????????????????0100??1??1100?000001210??10000000??????????????????????????????????1100100011001100100??????????????????????????????????????????????????????????????

Compsognathus_longipes ??00?011000????1010?1?00??1101000???0000???000000000??00001000000?0-0--111?0????1?1101???000?0-001000-0?0??????0???????????????0????????1????????????????010??0????????????????????0011000-?0?00-00-0-000100--0?000??1210?101000100210110010000000000100-0??0??1010-1?0?0?00010??0?1???0?10?0?200?00???????10??0010?103?????0??0000?0?000?01000010000-1?1000??????1??00020000?1110???000000?0??1???1?00??01???000000????0?01011???00?1000?1?22???12??10000011001??0-1000???0???0??0?0???????00?001111???00100001000100010100?0-?1??????000??1????1???00?0??????00?0??????0?0001000?10?001?0011000?00?0?0?02001000-

Huaxiagnathus_orientalis ??0???000001?0?10100??00??11000???11??0????0000?1000?????110?0000?0-0--?1?0????1???1?????????0-?0??0????0??????????????????????0?????????????????????????????????????????????????0?001?000-00?00-00-0-0???????????????????10??0??00?0?1-00100000000?0100-0??????0????????????11??10????0??0?0?100??????????10???01??10????????????0????00?0?120?1000??1?0000??????10000020000?1110?00000000100???????????00????00?00?0100?001???00?0?10?0?1?22???12111?000000?01?00-10001010???0??0?0??02??000?1011?????????000100000001010000-?1??????0?????????????0????????????0???0????00?2000110?001?0011?0??00?0???020??000-

Sinosauropteryx_prima 000000000001?001??0?0?????0-0000????0?0????0000???????????100????00-0--?1100????11????????0??0-00??????00??00012?0?10??????????0??????????????????????0???????0??????????????????0?001?000-00?0??0??0-0?0?????????????????10100?1002101-0?1000000?000100-0??????010-1?1?0?10?10??11????0?20???100?00???????1000?01???03?????????000?0?000??1120?10000-1?1110?????????00020000?1110000100001200?00?00?????01?0??00000?1100?0111110?1??11?010?22???121110100010001000-1000???0???0??0?001020?000?1011?????1???000100000011?100?0-?1??01?0001??0??0?1????0?01??10?00?0???0??0?00?20001101001?001100??00?0??0020??000-

Nqwebasaurus_thwazi ????????2??????1000??1011?0-00??00?1???----0??????????????????????0-10?1?2011101100201001??0????010?0-0???????0?00?10????????????????????????????010?00?101???0000-???????????????????????????????????????????????????????????????03?11-21211???????--00-0???????10-1??01200?0-?11111???100100??????????????????????????????????????????????????0???????0????????????1002100001?10000000??0100?100?0100?10??0?00000010?000000101001001000101221001111001010110???????????????????????????????????1???0?0????00??????????????????1??0??1?????000001000001011111?0010?0010?0?0002001110?001?0011000000001000?0101010

Anserimimus_planinychus ???????????????????????????????????????????????????????????????????????????????????????????????????????????????????????????????0??????????????????????????????0???????????????????????????????????????????????????????????1?????????????-?????????????????????????????????????????????????????????????????0????????????????????????????????????????????????0?????????111200000????0100000?012???????2???????????????10000?1010-01?1001001?002210??111110012210????10100?100?11?0???????1010000?0010????00???00?1000100010??000-???????????????????????????????????????????????????????001?1---?00?010110???0??1???

Archaeornithomimus_asiaticus ??????????????????????????????????????????????????????????????????????????????????????????????????????????????????????????????????????????????????????????????????????????????????????????????????????0?????????????????????????????????????????????????????????00----20220011000121100000000020000000010000000000010031?1010?0?1000020000000?00??0?????0????????????101210000111001?1000?00200100012000010000000000????001010-11?100110110022??011???00011210010?0-0000100010000100000100010??00101100011-01001010100010000010010100100011011000100000101111100010?0????100002000110100101---100010011000?0??1?1?

Beishanlong_grandis ????????????????????????????????????????????????????????????????????????????????????????????????????????????????????????????????????????????????????????????????????????????????????????????????????????????????????????????????????????????????????????????????????????????????????1????????????????????????????????????????????????????????????????????1???????????1112100001?00010000?0002????000100000000????000??????????????????????????????????000002?0?????????????????????????????????????????????????????1????????????10?0??00021?0110?10001010???1100010?0?1010100?1000110???1?001?????00??10????001011

Gallimimus_bullatus ??0???00210000000101?101110-0?000???000-???00000000?11001?110?000?0-0--1???111011200010000?0?0-?000?0-001?010112??00-??01?010?10?0?110??1?0000100010?0001001??00011??110?0-100010??10-0??10?1010-00-0-0?01?10?1?1000-0???011------1---??-?-?-2---???---?????10??010-??202210010??12?????0?0?????????00?1??000??0??0?0040?1??0???100?0??00??0??0?01?0????0??0?????????011200000??10?1??0000?02?0?0???2??0000??0000??1?0??001010-?10????????0?2????0??2?00011010?1?010100?100011?0??0????1010000?0010??01001-?000100010001010000-110?????00?1001?0????00?00??????00???00?0?0100??0001???00101---100?01?1?010?0001010

Garudimimus_brevipes ??0000002?000001001?0101110-00000111000----0?0000000??01-0110000010-0--111011101120?0100-000?0-00?000-00?00?00?001?113?01?01001000?11?0????00010001?000010?01100?11?????????0???00???-0001001?10-00-000?00?1001?1000-0?1??11------1----------2------------000?-1?????????????0-???2???000001001000001001???000000001?040?10100?0??0000???00?0??0??0???????????????????????????????????????????????????????????????????????????????????????????????????????????010011100010011100?10010010010001001001000101020??????????????????10101100011001000110010001011100000?0100?010002000110?00100011100000?1101020000011

Harpymimus_okladnikovi ??00000021000?010?0??1011?0-000?0?11100----000000000????????0???????0--1110111?112010?00-000?0-00?000-00100100?????????????????0?????????????????????????????????????????????????0?0010001001011000-0000010100??0000000-0011------1------?--11?0??0?--?---?????1010-???0??0000-??02?0???0001?02??000?00????00??110?11040?1?10???1000020000?0000001????0?0110??????????11??????1?10?1?100?0?12?0?000120000010000000011?10000010-10010011001102210002111000111100100101001????11?0??0??00100?000000????010????00???????????????????0101?00???0?????????????????????10????????0001000110?00101---100000?0?000?0001011

Ornithomimus_edmontonicus ??0?????210000???11???01????0?020?????????????00???011?0111100000?0-0--????1111112?0010000???0-?00?????010010101??011??????????0?0?11???1?00?????0???000?0?0??0?0???1110?0-10?010??10-0??10?1010-00-00??01?10?1?111??0???011------1?-???-?-??2??????????????10-?010-?1??22100???102?????00??00???0??1??1??0?0??0??0?0040?1?00???100?0??0???0??0?01?00-??0??0?????????11121000???00?10?0000?02?0?????2?????0??0000??1?0?0001??0-?1?????????0?22???0??2?00012210???010100?10001100???????1010000?0010??010????00010??10001010000-110?????00?1001?0?10?0???0??????00???0??0?0100??0001???001?????100?01?1?0?0?0?01000

Pelecanimimus_polyodon ??00000021000101000111011?0-00010011100-???00000?00000001111?000??0-0--1120111011202?1?0-000?0-00???????????????0?010?00???1??10????????1?00?01????????????1???01????1?????10????0??00?001000?1??00-0-00010100??1?0??0????1030001103201-102110201010--10-0???101010-1?????00010?1???1?0000?1??????00?????????????????0??????????????????????????00?110??????000001???1????????1?10?10000?0??200?0001?000?0100?0000011??00?1010-01?10?110110?221000112100011210?????????????????????????????????????????????????????????????0??????????????????????????????????????????????????????????????????????????????????????

Shenzhousaurus_orientalis ??00000021000001000??1011?0-000?0011000?---0000010001?????1???????0-0--1120111?1????0?00?0?0?????00?0-0???0?01????0????0???1???0??????????????????????????????0??????????????????0?001?001000010-00-0-000??1??1?0????0?????1------1---1-----11001001--00-0?????????????????0???????????0?00???200?000??????00???0001?041?1??0??????000?0000?0??0???0??0?01???????????????????????????????????????????????????????????????????????????01??10?2210???1210001011001000-100010101000???1000100?00010010010100?1?010100000000010000-???1????001100??00100??????????????????????????????????????????????????????????????

Struthiomimus_altus ??0???0021000??1?10?110?110-0000011?000----?0000100?11???1110?00010-0--11?01111?120001000100?0-?000?0-0010010112?1011??01?0???1??0????0?1000?????0??0000??0???0?010??110?0-100010?000-0001001011000-00??01010?1?1010-0???011--?-??1----------2------------??10-?010-?????2100???002?????000??????0?????1??000??0??0?0040?1?00???100?0??00??0??1?01?0????0?10?????????111210000??10010?000000200100012000001000000001?0?0001010-01010111011002210001?2?000112100100101???10001100?10????1010000000100100001-0000100010001010000-110?01?00011001?0010?0?000???1100010?0?10?0100?200011??00101---100?01011010?0001010

Achillesaurus_manazzonei ???????????????????????????????????????????????????????????????????????????????????????????????????????????????????????????????????????????????????????????????????????????????????????????????????????????????????????????????????????????????????????????????????????????????????????????????????????????????????????0?2?0??00??1121???10???????????0?0?????????????????????????????????????????????????????????????????????????????????????????????????????010???????0--?01102?0????111????0??????????????????????????????????????211???0?????????????????????10????????100{12}001110?00??????0??0?0?010??????????

Albertonykus_borealis ??????????????????????????????????????????????????????????????????????????????????????????????????????????????????????????????????????????????????????????????????????????????????????????????????????????????????????????????????????????????????????????????????????????????????????????????????????????????????????????0??????????????????????????????????????????????????????????????????????????????02101011??1????????????????????????????1?????1?011?12???????????????????????????????????????????????????????????????????????????????????????000?111111001????????????????????????????????02-01????????0??

Albinykus_baatar ??????????????????????????????????????????????????????????????????????????????????????????????????????????????????????????????????????????????????????????????????????????????????????????????????????????????????????????????????????????????????????????????????????????????????????????????????????????????????????????????????????????????????????????????????????????????????????????????????????????????????????????????????????????????????????????????01????????????1??12?0???????1?00??????????????????????????????????1????????????????????????????????01???10010?11?11111??11100011000002?110102010000-

Alvarezsaurus_calvoi ?????????????????????????????????????????????????????????????????????????????????????????????????????????????????????????????????????????????????????????????????????????????????????????????????????????????????????????????????????????????????????????????????0---?00?0?0?0-00121011000?1??????????????????????????30020????????12100?0?1002???????????1???????????000-00001?0010??000?0???????????????????????????????????????????????????????????1?011?1101000-10020--0110021002001101?00???????????????????????????????????00?11?1??001????????????????????20????????1011??11111001??????00?00?01110??10000-

Bannykus_wulatensis ????????????????????????????????????????????????????????????????????????????????????????????????????????????????????????????????????????1?01???????????????00-0001????????????????????1?????????????????01011????????????????????????????????????????????????????1{01}01??111010100?1?111????1????????????????????????????0??0?1????001??00???1?00?0100??0100????????????111100001?0?0010001012000002100101102110110?0?1?11000111110111011111002201112???01000111010???????????0010010????11000000?????????????????????????????????11000100010000000111000101111110020110??00000010?111?10010????1000000010002?01????

Bonapartenykus_ultimus ???????????????????????????????????????????????????????????????????????????????????????????????????????????????????????????????????????????????????????????????????????????????????????????????????????????????????????????????????????????????????????????????????????????????????????0?0??00?0???????100110101101??????????????????????????????????????????????????1110-11101?01001?001??????????????????????????????????????????????????????????????????????1????????110????????????????????1111??0?1??????????????????????????????12??????????????????????????????????????????????????????????????????????????

Ceratonykus_oculatus ??????????????????????????????????????????????????????????11?01-1?0????1110111?1120????0?100?0-00??????100??0?????10-??????????0?021????0?-001?????????????00-0000-?1??????????????10????????????0??0-0????????????????????????????????????????????????????????????????????1???0?221??1???????????????????????????????????????????01?1??????????????????????1011?????1?00-0000??????????????0????10????????????????????????1?????0?????????0???11?2?????????????????????????????????????????????1???????????????????????????????1???????????0???????????????????????????????11211?110??010?????00102-?1001????????

Haplocheirus_sollers ??00??0011011111010111001110000100111000?0?0000100001?01-1100000000-0--1000111?112010000-10000-00??00??010011000??010?00110100000021?0001?0001000010?0111??00-00110??????1?110??00?1011110-0000100100-0001111010001??02100101000100120110010001101000100-0??????00-?--101?00?10?10111?101001001??0100001??1??0??1?101030?00?0?0?{01}?00??00100?00?00000??1?01???????????110200000111?100000101100110110110100?00?1100001?1000000101011011101100220111212101010001010???????11-?01002?000001001??000011010100??01001100000110110?0-01??0110001000000?1010001011110?00?011010??000?20???1?10010?????00?00?010002001000-

Kol_ghuva ??????????????????????????????????????????????????????????????????????????????????????????????????????????????????????????????????????????????????????????????????????????????????????????????????????????????????????????????????????????????????????????????????????????????????????????????????????????????????????????????????????????????????????????????????????????????????????????????????????????????????????????????????????????????????????????????????????????????????????????????????????????????????????????????????????????????????????????????????????????????????????001?0011000002-?11112010?0??

Linhenykus_monodactylus ?????????????????????????????????????????????????????????????????????????????????????????????????????????????????????????????????????????????????????????????????????????????????????????????????????????????????????????????????????????????????????????????????10-1??0??111????1210????121212????????????00???0?11?0?{01}?20?10??10?121?00???0020????????1???1011?0??????????????????????????????????????????????????1?10101111010111111-210032111?2???11011?120??????????????????????????????????????????????????????????????????0??1?1???0010???11??0????0111???11?????????112??0?1?1?01?00110?0002-?1000?????0??

Mononykus_olecranus ?????????????????????????????????????????????????????????????????????????????????????????????????????????????????????????????????????????????????????1100?????????????1001011??0??1???????????????????????????????????????????????????1-212?1??0????--10-0???????0-?--1010?11????1211?101021012001000000-?10100???1010???2??1?????0121?0?1??????????????????101100???1000-00001?11001100000230000101111-0021000111011?10101111010110101111002211112-?111011?120?0?0-???????????????1?00111??00?310---???1???00?0-0000??-?????0-?10011112-20010010111000011011110011?11???1011??1101101001?0011000102-11000?010100-

Nemegtonykus_citus ?????????????????????????????????????????????????????????????????????????????????????????????????????????????????????????????????????????????????????????????????????????????????????????????????????????????????????????????????????????????????????????????????????????????????????????02?00100?00???0-01??0????00???01??????0??012100{01}1000{12}0????0??0?01???????????1000-00001100000?010?0???????????????????????????????????????????????????????????????????0100?????20--00000210210010110???3????????????????????????????????1?0111????0000??011100001111111001110?10???111111101011010????0001?????000????0?0-

Parvicursor_remotus ?????????????????????????????????????????????????????????????????????????????????????????????????????????????????????????????????????????????????????????????????????????????????????????????????????????????????????????????????????????????????????????????????????????????????????????{01}2??12?0?00???0?????00??010????1???1?????0121???10?????????????????????????????????????????????????????????????????????????????????????????????????????????????????????0???????0--?????2?01?0?0-?1????3?0---1---1--0000-0000----1{12}00???1?011112-200100101110000101111100011000?01?101211011010010????000102-11001?001100-

Patagonykus_puertai ??????????????????????????????????????????????????????????????????????????????????????????????????????????????????????????????????????????????????????????????0????????????????????1???????????????????????????????????????????????????????????????????????????????????????????????????0?00?001?0?01???10000?01???1???30?2011000??012100?002?????????????????????0???1000-1110????????001????0??011?1111002010011???????00?10??101?????????1??11112?????011?11010???????110???????00100111????02?11011---01001???????????????0-0??????1112000100011100001111111??10111??000010{12}001100110??????1????000??????010?0-

Qiupanykus_zhangi ?????????????????????????????????????????????????????????????????????????????????????????????????????????????????????????????????????????????????????????????????????????????????????????????????????????????????????????????????????????????????????????????????0-?-?0???0?10-??1????????????????????????????????????4002??????{01}?012100?0100?0???????????????????????????????????????????????????????????????????????????????????????????????????????????????0?????????0--?001????2100111????0?1????1---???00??????????????????1?011112-20010000111?0001101110001?100???1?101?1???1010010????000102-11000????0?0-

Shuvuuia_deserti ??000000110000010101?101102100000?11000-???00000000011000-11001-1?100--12201110112020000-10000-001000-0100000000??10-??01?1200000021110101--01001011111?00100-0010-??11001011110001100?001000001000-0-0000-0-010011110??001????0??03201-212110202?10--10-0??10-??10-10100211110?1121011?102?01???000???0??10000?001?1050?20?1?0010012100110100200101????1110101100???1000-0000110?001000000230000101?11-002????110?11?101011110-011010112--02211112---1101121201000-10020--0111021012001111000?310---1--11-00000-0000----22000-010011112-200100101???0??1???101?001??????101112110110100100011000102-11000?011100-

Tugulusaurus_faciles ???????????????????????????????????????????????????????????????????????????????????????????????????????????????????????????????????????????????????????????????????????????????????????????????????????????????????????????????????????????????????????????????????????????????????????????????????????????????????????????????????0??00??????0?????????????????????????????????????????????????????????????????????????00?1111101????????????011?2???0?000?01??????????????????????????????????????????????????????????????????10100000???0000001010001011111???20????????001010?1101????????????????????????????

Xixianykus_zhangi ???????????????????????????????????????????????????????????????????????????????????????????????????????????????????????????????????????????????????????????????????????????????????????????????????????????????????????????????????????????????????????????????????????????????????????0??2?01??0??????0-0100???00100050?2211?10???101????????????????????????????????????????????????????????????????????????????????????????????????????????????????????????01000-?0020--0110??10110110111???310---1---0100000-0000----220?10010010112-20010010111000010111110011???100101011111110?1110????0?00?2-?10?0????????

Xiyunykus_pengi ????????????????????????????????????????????????????????????????????????????????????1010?????????????????????????????20???0?????00201101100111011110?00110001000011?????1?0110?0??1??????????????010??00010110100100-????????????????????????02???0????????????1?1{01}010001101010001?1?11000100010?0?11001001???1???1100????????????001000?000000??????????????????????111100000110?10010010110010020001011?21101?????????????????????????????????????????????????????????????????????????????????????????????????????????????????1????????????0000101000101111110020110??000?0??1?????1001?001100?0000010????0?0?0-

Alxasaurus_elesitaiensis ???????????????????????????????????????0?????????????????????????????????????????????????????????????????????????????????????????????????????????????????????????????????????????????1?00110101??10-101?0?????????????????????????????00110?102110001010?0?????????????????0???????????0?00?001001??0011??000??01001004??1??0??????????01??00?2?01??????0000??????????0????????????0?????0?1000100111001101????00??0?100010000-00?0??1100101220??02???00100100???110100?110101?0???????0-??000?20???????1?????011110?1?1?220?0-???????111?00???0??0?10???????????????1?????????????????00?010-?00?00???0?0?001?0??

Beipiaosaurus_inexpectus ??????????????????????????????????????????????????????????????????????????????????????????????????????????????????????????????????????????????????????????????0??????????????????0???1?00???1?1??10-101??100-?????????????11------0220001100132010001010-0???????????????????????1?????0????????0????????????????????0?????????????????????????????0???????????????001002000001?10?0?000?0?100??????????10????????0????00100?????0?0??????0?22??????1?00?0000?01011110011??????1????????????????????????0???????????????0???????1??????00???0?0?0??0????0?????010????????10?0??0??????001???11??????????????????0-

Erlikosaurus_andrewsi ??100000100110000?-0?101110-00001101000????0?00????11????1000001010-0--?1100--?110?10000010000-00100??0000010000?0??0???1012100001---01?0????00?00?1000000000-??00-????????10??000?1010?011?1011010-101000-0-11?0000-0100011-?----0220001?0013111?001010-???????????????????????????????????????????????????????????????????????????1??????????????????????????????????????????????????????10?????????????????????????????????????????????????????????????????????????????????????????????????????????????????????????????????????????????????????????????????????????????????????????0????????0??0??????????0????

Falcarius_utahensis ??????????????????????0???????????010??001?0???????????????????????????????????????100000?0?00-00??0??????????1?10010???????????????1?00??00?00??1?01000?000??0?011?110-10011010??0??1?00100000??00-1010??????????????????????????0?20001{01}00101110001110-0???????1101?212100010?11211001120100200100110100101010?0010041?101000?1000120010011000010???101111?????010110020001?1?100001001?11001?00100001111001100?000110010010-1010001100110220000211100100000010111100111010101?00000011010??11010010101010001121023001022000-010?01101011000000100000101111100010?11??010000100011010001001100??001000???001000-

Nothronychus_graffami ??????????????????????????????????????????????????????????????????????????????????????????????????????????????????????????????????????????????????????????????????????????????????????????????????????????????????????????????????????????????????????????????????????212?????????2??????20?00?001??111?????????10???04??10?0?0?2010??10?0??0?000100?????00???????10100?2????00???0?0100000110?1?1111001??100?100000?????????0-????0????????22?????????000000001011120011??1???1?0??100???1100?10101000111--2?0121?0?11102?10???0??112111100??00?000?0010110???111??1101?0??0?2000?1??0001010-0000001?0110?0?0??0-

Segnosaurus_galboensis ??????????????????????????????????????????????????????????????????????????????????????????????????????????????????????????????????????????????????????????????1????????????????????0010?????1?1??1??101??0-0-?1?000??0???0????????0???001?0?10111?00101???????????????????????????2??????????????????????????????????????1??0????????????????????????????????????????2??????????10?????1???11?0?0??1100110??????????????????????????????????22????????00???????1011110011102010000101000-01000?201010????01000011110?1?102?101??0??????????????0?0??1??0???????11???11?????0??10000????001010-?00000??01102001????

Nothronychus_mckinleyi ?????????????????????????????????????????????????????????????????????????????????????????????????????????????????????????????????????????????????0?1??00?0?0??1??????1??1??10?10??0???????????????????????????????????????????????0???00110?101?1???1010-0???????110??2??010011?11?????112010??001???11100100??010??00????????????100????0????????????????????????10??????????1?0??0?110?0?1101?011110?1101000100??0????????????0?????????0??????????????0000????1????????????0???????????????12???????????????12110?111021100-00???????????0????????0??0??????11??111?1?10?????????????????0-?0??????????????????

Avimimus_portentosus ??????????1?1?????????????????????????????????????????????11??1?????????????????????0000?????0-?01??????0?00?000???11????????????1?1?0??1?10?00??01??120?0?0??0?00-?110-????????0??00????11?????????11??10-0-?0?00???????111-??-??????????2??2????-?--??????110?111010012000110?11211100100100100000111100000?0000011060?1010?0?2?0000???00??????????????????????????200200000110010000100011001001010000100100000001?111????0-????1??????0??2????????????????0100??1???1000011101010001111?00?00100100011-00011110230010?0000-011011210110100000101000001001100011?11100010112000110111100?11000102-110102010100-

Caudipteryx_zhoui 100000??0?111?0?0000????????0?0?0?11??????????00???01?????000?01?0??????1?0101?10-?10000010??0-00??0???00????000??0????????????1??????????????????????????????0??????????????????0?201?001101111100-110?1??0-???0?0?????0?10???1101?--??-12??2????01--?0-???????0?0-????1??00??????????0?{01}0????00????????????????????04??1??0???2100??11????0??00?0???1?011?0?00??11000???????1?00?001?0?0?????1??10?00??0000?0??0001?110?0000-?0?1?111?010?22???021-100?000000100101001???0?1?10?0?000?????00?????????01???2011110??00102?0?0-?100?111111?0?????10??00?????????011???10????01200011?1001??0????01?1?0???1?0??000-

Chirostenotes_pergracilis ??1????????1?00??????1101??????????????-?????????????????????????????????????????????????????????????????????????????????????????1?0100???01?1???0???120?011??0?00-1?110??????1????10-0?01111111100-100-10-0-?0??00??0????????????1?--????-??2????-??????????????110??211??0?????12?????1????????0??10?1??1???????????52?10????0??1?0?????????????0??????????????????20?200000?????0??10????????????????????????????????????????00?0????0???22000???????10000??1000-100010000??1??010000-000000??12??00?????201111023001121000-01????0?0??????0?0?11??0?0??????00??????????00?????1???0?1??0???00?01?010?0?0??00??

Citipati_osmolskae ??1000000010100101?0?110100-00020111000----0000100101?0010100001000-1101100101?10-1111000100?0-001200-000?000000??010?011011111101?0??001?01010?1010112000000-0?001??11111110?0000021-0201101111100-110-10-0-1010001100-0111------1----------2------------??10-?110-?????210010??1??????1?0?10?????????1??100??0??0?1?52????0???10?????1???0??2?01?1????0??0010011111200????????00?0??1100?10?1?????1?000?0???000??0?1?10?00????0?????????0?22???0????0010000?????0-100????001?1???????0-01000?1010??0110???20?11102300102?000-01??????2110010?0?10?????0????????????????0000??000????001??0???00?00?0?0?0?0??000-

Conchoraptor_gracilis ???0????0011100?0?00??1?????0?020??????-??????01????1????110??010?0-???????101?10-?1000001???0-?01??????0?000000??0?????????1??1?????????????0??????0?2???1?????00-??110????????0??21-0?01101??1100-110-1??0-?0?000??0???111-??-??1?--????-??2????-????????????????????????0????????????1???????????????????0??1??0????1?1??0???????1??????????????????????0????1???12?020000???00????0100??0?1????????00?0???0?????????????????0?????????0?????0?????00100100????0-100?11?001?1???????0-10000?1010??00?1???20???1????010??000-0???????11?001??0?10?????0???????????????????0??????????01??0???00?00?0???0?0??0???

Gigantoraptor_erlianensis ???????????????????????????????????????????????????????????????????????????????????????????????????????????????????????????????????????????????????????????????????????????????????20-0001111011100-110-1??0-00?00?0-????????????????-???-??-2------????????????????????????????????????????????????????????????????????????????101012?0000?0??0??????????1???????????????????1?00??0100???110?110002?0??00-00000000?????00010-10?000??????????????????????????????????????????????????????????????????????????????????????????????111110????10001??????????????????????????????????????1?????????????????????0?0-

Incisivosaurus_gauthieri ??1000000101110100000100110-000111110001???0000100001100010000?1000-1001200001000-101000010000-00100100000000010010102011012101101-000001?0110???11?012001000-?0010?11??1100-??0?0?2011?01111111000-0-0000-0-00?0?0110??0?101200?00?00??1?2001101?10--10-??????????????????????????????????????????????????????????????????????????????????????????????????????????????????????????????????????????????????????????????????????????????????????????????????????????????????0?????????????????0??????????????????????????????????????????????????????????????????????????????????????????????????????????1?????????

Microvenator_celer ???????????????????????????????????????????????????????????????????????????????????????????????????????????????????????????????????????????????????????????????????????????????????{12}0????11?1101100-????00-0-0????????????????????0???????2??2????-?--??????1101?110???????0010?01?1?11102??10100000?00100100010??0?10?0????????????1?11??00??2??????????????????????2002???????00?0??0?0??010110??02?00010000?000?0???????0????0?????????????????2???0010?00001000-1001101001???1010000-00?00100100?0101???20????????0?????????1??11?10121010000100??000???11?001??001000000120001101??????????????????????????0-

Oviraptor_philoceratops ???100??0?11?????1????10????0?0?0??10??-???0??01????1????01000???00-????1?0101?????1?0?0??0??0-001?0????0??0??00???10???1?1?1??1?1?0?1?????????????????????0????00-??????????????0?21-??011?1111100-110-10-0-?0?00???0????11-?????1?--??-?-??2????-?????????????????????????????????????????1??????????????0????????????????0???????????????????????10???????????1111??????????????0???1???1??0????????00?0???0????0???1????????0?????????0??????0????00?0000?????0-1???????0??1???????????????????????????????????????????????????????????????0?10?????0??????????????????????????????01?????????????????????????

Rinchenia_mongoliensis ???1?????011100?0?00??1?????????????0??-??????01????1????0100?010?0-10?????101?0????0?00?????0-?0??????00?000?00????????1???1??1???????????0???????????0???????????????????????????21-0?011?11111?0-??0-10-0-?0?0?0??0???111-??-??1?--??-?-??2????-?????????????????????????????????????????????????????????????????????????0??????????????????????1?????????????1??1??????????????0???1?0??0?0???????????0???0????????1??????????????????????????????00?000??????102???111?01?1???????????????????????????????????????????????????????????????0????????0??????????????????????????????0???????????????????0??????

Buitreraptor_gonzalezorum ?????????0000??1?10???0?1???0??????????1???????0???????????00????0??????????????????1?10010??0-001?0????0???0?10?001???????????????????????????????????????0?????????????????????????1?0?0-?0????01010????????????????????????????0??01-0020???00?00??00-0??110?010-??201210110??12????010??????????0??1????0?????1?1??0?1??0???????1??01??10?2?01??????1??2???????102?1???????1100???101101001???????????0??1??????1?110???????????????????22????????????????0100??????????01?10?0????111?111?10????010????20112122??0112?0?1001???????1??1??????????????????????1????????11?20001????01?0011?10??1-01001?0???1??

Deinonychus_antirrhopus ??000000200001?111101000?00-000000010001000000010000110??1000001010-0--1???100?110??10???10??0-00??0???1001000?0??11031?110100?0???????????1?????????00??0????0?0??????????10??????0011?00-?000??00-0-0001010?1001???110101011000002100?001000000?000100-0001111?10-00101200011?01011010020100000000000100101011101100??????0?????00120010010?1001?1????1?12?????1???21120000??110?0??1010?10?1?0???1000010001000?00?11100001101001001100111220000212?0010000001?00-1??1100001?1??0?0000-1110012010??0101???001111222001021010-0101011111111100001000001011111?00101??10?000002000110100110011?10?100011101001010-

Dromaeosaurus_albertensis ????????2?0??1??1?1??100????0???0?010??10100??0????011???100??0101??????1???????0-1210111???????0??0?????????000??01031???0?0??0001000001?00000100?0?0011?01010000-1110-10-100000000011000-00000-00-0-000100-111010111101010?000110?000?00000000000?0100-0???????????0???????????????????????????????????????????0?????????????????????????????????????????????????????????????????????????????????????????????????????????????????????????????????????????????????????????????1??????????????????????????????????????????????????????????????????????????????????????????????????????????????????????????????010-

Linheraptor_exquisitus ???000002000?1?1101?1?00?10-00000001000????000?0100???0001000101010-???1110100?10-1?1000?100?0-00?200-00001010?0?0?10????????0000?????????????????1?10101????????????????????????0??0??000-000???00-0-0?010101????0??111???01?????02101?0?1000000??00100-???1111010-1??00?0??11?11??1??????????????????????00???????10??????????10?0???000??001000?1101???1?1110??????????????1?????01?????10?11???01?0??1?????10??????????????????0???00?????????2????????????1?0?????????????????????????????20????010????01??????????????????1???????????????????00?101??111??10???10???0??2000110??011001??10?10?0?10???01010-

Mahakala_omnogovae ???????????????????????????????????????????????????????????????????????????????????100?00???????????????????????????????????????0???????????????????1001??1???????????10?0-10?????????????????????????????????????????????????????????????????????????????????????????????????????????????????????????????????????????40?1??0???????1??01??10?2?????????1????????????????????????????????????????????????1???10??????1110?0????????????????????????????????????1000-0???0-??11?1???????0-11100???????????????????????????????????????1?11?111?0?0???0?0????????00??????0???00??0001????01?00??010?10-?1??0?0??010-

Microraptor_gui 100????01000???1??????00??0-0??????????????00000???0?????1?0?0??????????1?0??????????0????0??0-00??????????????????????????????0?????????????????????????????????????????????????????10?0???000??00-0-0?????????01???1????10???01?02?0??0?10000???00--10-?????????????????0?111??1??????00??0?1???????????100???0?1?004????1????10??1?001??1--1-01?1??????120100?1110?0?????????100???1111010?11???0????110??????000?110000000-10?10?0????0?22???02??1001000000100101?0?111??1?1???????0-011?1?200--?010?1-0011121?2??011011010?1??????11?111????10???0?0??????00??????0?0?0???0001???10110011?10?11?0??01?101010-

Saurornitholestes_langstoni ???????????????????????????????????????1??????0??????????1??????????????????????????1?111?????????????????????????????1?????????????????????????????????????????0???????????????0????1??00-?0?????????0????0-???????????1????1???0???0??0?1000000?000100-???1111010-1?1??200011?11?1011?12??000???00???1??10100110110?41?1010100????1??0???1??2?01?1????1??1?????????20??????????????????????0?????1???00?0?00?1?000??1?0?00?0-100???110011022??002???00100000?10?10210110100??1?0010011100100???????????????0???1????0?0??0??????????????????????????01011111???101??1?01100110001100101?0011?10?10?001?0?001?10-

Sinornithosaurus_millenii ?000??001?000??1111?1000??0-00001?11??01???0?00010????0000000?010?0-0--1??0100??101110100100?0-001201101?0?0??0??0010??????????0??????????????0??????????????????????????????????0?101?000-00000-00-0-0?010100??0?0??1????101010000?10010?1?00000?000100-0???????????????????????11?????1????????????????????????????04?????0???????1??0??????2?????????????01001111?20120000???10?0??1011?10?????????????0????????0????0000????1?????????1?22???02?2?00?0000?????0-1???11??01?0???????0-11??0?2011?????1???011121????011211010?1??????????1????????????????????????????????0??1001???001?00???10??1?0???1?001?10-

Tsaagan_mangas ??00000020000?011010?100110-000000010001???00000100?1?0001000101010-0--1100?????0-1110001100?0-001200-000010?000?00103????0?00000010010010010001011010101100???0000??10110-1000000?001?000-00000-00-0-0?0101011101011111?010101?0002101?0?1000000?000100-??????????????00?0?????110??01?????????????????????????????????????????????????????????????????????????????????????????????????1?????????????????????????????????????????????????????????????????????????????????????????????????????????????????????????????????????????????????????????????????????????????????????????????????????????????????????????

Unenlagia_plus_Neuquenraptor ??????????????????????????????????????????????????????????????????????????????????????????????????????????????????????????????????????????????????????????????0??????????????????????1????????????????????????????????????????????????????????????0??????????????????????????????????????20?00101??????1??101??11011?0?0?1??0?0???????????????????????1???????????????????????110000?110?00100110211?????????????????????????????????????????????????????00???01000-210011100111??010000-??111?20110101011-00011110??0011????11010?01?1112101000?10??00?????11??01??????????????????????1???????0????11??1????????

Velociraptor_mongoliensis ??00000020000?0111101100110-000000110001???000001000110000?00101010-0--1120100?10-1110110100?0-000?0???00?10?000?0010?1?1?0100?00??0010??001?00??1?01001?000??0?00-??11110-100000010011000-00000-00-0-000100-?1?0?0111101010?110000?00110010000000000100-0??111?010-???012000????10??0101???10?00????0?100101??1?01?0050?1010?0010001??010?1????01?110??1?1201000110020120000??100000?1110010?1?0??01?00010??1100??0?1?10000????00100?????0?2200002?2?0010000001000-110011100101?00?1000-01100020110?01011-0001111022001021010-0111??1?11?11?000010000010?1???100???0????0000?20001???101?0011110010001111?001010-

Epidexipteryx_hui ?-?????????????????????????????????????-??????00???0???????0?001?00-???????????????0???00?0??0-001??10????????00???0????????????????????????????????????????????????????????????10?2?0?00???1?10-00-10001??0?01?00???????010?011100?01??2120?101?0?1??00-0??????0????????????????12????000????100??????????00???0????04???0?0?0?2100???020?1??2??100????????1?0??????2?1????????00??0100?1?01?02?????0?0010?0?0??001????????0???????????0???22???0??????00001?010?0-200011??????????0???????0??0?0??????????0?010102?????110????1????????????????????0????????????0?????????1??0001???111000110????0?0???0????????

Epidendrosaurus_ninchengensis ??????????????????????????????????????????????0????????????????????????????????????1???0????????01?????????????????????????????????????????????????????????????????????????????????????0011111?0-00-100?10-0?????????????????????????????1???1?1?0?1?????????????????????????????1???????0????1?????????????0?????0?1?????????????????????????1???????????1??????????2???????????????100?1?00??2?????????10?0??0?000????????0???1??01???0???22???0??00?000001???????????????????????????????????????????????????????????????????1????????????????????0??0?????????0???????????????????????001100??00???????0???0??

Scansoriopteryx_heilmanni 0?????????????????????????????????????????????????????0???10?001000-???1???????1?????????0??????0????????????????????????????????????????????????????????????????????????????????0?20??00??01?11-00-100?10-0?0??1?0??0????1????????????????????????????????????????????????????????????0??0?0?100??????????00???0????030???00?0?10?0??001??10-0-??0???1??11??????????2??2000??1??????0?0?1?01??2???1?0?0?00???0000001?11000011010?10100?0?0?220000210000100000?10?0-2001????01?1?00????????????0?0-?????????{01}00??0?2?????1100???1??????2-???1????1????????????????0????????????????1???0??0011?00?00???00??101?0??

Jinfengopteryx_elegans ????????1?01???1?10?0?????????001??????????????0???????????????????????????1??????????00????????0?0????????????2??0??????????????????????????????????100????????????????????????????0????0-?0??0-0????????????1??????1?????0?000??0???????????1???????0?????????1????????????????????????????????????????????????????0??????????10?????02????????0?011?????2?????????2?1????????1?????1010????????????????????????????110?0?????????????????22?????????0?0?????????????????????1???????????????3????????????0?????????????????????????????????????????????????????0???????????????????????????????????????????????

EK_troodontid ??????????????????????????????????????????????????????????????????????????????????????????????????????????????1????11??????????0????????0?????????????????10????1????1??0?????01?????????????????????????100-???0????1???????????????????????0???????1???????0-????????????????????????????????????????????????????????????????????????????????????????????????????????????????????????????????????????????????????????1000010-?0?1???????1?22?00?2???0000??????????????????????????????????????????????0?????????????????????????????????????????????????????????????????????????????????0011?00?1????0???1???10-

Byronosaurus_jaffeei ??000000110000010001?100110-000001110001????00001000??0000???0????110--11101110?0-?0???????????????????????????????????????????000?11?0??1???0?00011?100111????010-??11001010111?????0?000-00000-0110-0?010???????????????101?001002201-002000120000--10-0??10-?????????2??0??????210???1??????????????1??1???????0????????????????????0???2????????????????????????????????????????????????????????????????????????????????????????????????????????????????????????????????????????????????????????????1???????????????????????????????1???????????0?0?01??11???????????0?????????????????????????????????????1??

Mei_long ??00000011011?00?-??0?00?12100000?11000?????00001000??01--11?0?11?0-0--12?01110?0-1100?00??0???001100-0???????10???11??????????0?????????????????????110??????0???????????????0??0??????00-0000??0110-0???????100????1????10?0?0?001201-0020???0???0--?0-0??110000-?--101210110??1010??01?0?1?1000???00????00???00111040???1??0?1000?0001??2--0-0?0?1?0???12?????111120120000?1100000110100000010?111000110?0?????0???????????????10???00???2??000?1210010000001????????0--00??1?0011000-01100000????01001-0101??10???0??20??10?10?01??1110110?0?11?000?????????011?0?100??0112000110?00100011???1?1??10112101010-

Saurornithoides_mongoliensis ??1000??1100000??10?01?0????0???0?01???01000???0?????????1??????????????110??????????0???????0-00??0????????????????????1?01????00?11?????????1?????????????0-0??0-????????0-?11?0?000?0?100000??011??0?0????????????1?????0?000??02101-0010001000001010-0???????????????????????1?????0?00??????0??000?00??0???000??0?0?10?000???001????00???????????????????????????????????????????????????????????????????????????????????????????????????????????0??????????????????????????????????????????????01?1???0??111023001020000-0???1111111011??????????????????????????????????????????01??????????1???0???1???1??

Sinornithoides_youngi ????????1?000???????????1???0?00??????????????00??????????????01????????????????0-??????????????0??????10?????????0???????????????????????????????????????????0???????????????1????000??00-?000??0110-0????????????????????0?000??0?101???1??0100?10?010-????????10-???0??101???????????1????????????????????????????0??????0???10??1??01??1??0??1?0????1?12?????1???20120000???00?0??1??0?01???????2??0?10????????????10?0010-?0?1?????????22???02????0?0000??????????????001?1???????0-1???0?00??????01???00?1210??00?0210?0-?1???????1?011???????00?????????00??????0???0???????????0100011?0?10????011?101010-

Sinovenator_changii ??1???0001010??0010?1100111000?11???000??????000???011????????0?????????????????0-???0?00???????01????????00??12??011??????????00010110001--010??01101011110110010-?1110010101001??1?0??0???000??011100?0??0-?1?0?0??????0101?00??0?2?1?0?10?010??0?01?0-?????????10???0?210?10??01?????00???????0000?01??10000???0?1?40?1?00???10??1??01??2??0????????????1?????????20120000???10000?101?0????????????????????????0???10?001????????????????????????????0000???????????111????1???????0-11?00?2011??010????0011210??0011210010?10?01?11110110?0?11?00??11?111?0011???1000?10??0001???001??????00?10???1?1?1???1??

“Troodon_formosus” ??0???001?0?0?010?011100?10-0000?1??0??1??????00???0????????00????110--????100??0-?00?0001??00-?01??0-0???0?0?1???01030??1?11??0?00110000???001??010?1000001???010-1110-10-10?111??1?1??010?100??0110-00?????????0????????101?00?00?201?0?1000220?001010-????111?10-111??210110?01?10???1001001????????1??101??1??0?1050?1?10?0????011?0?0?20?0??1?????????2??????????????????1?00000110??0?0?1?0?10???01?0????????0????000010-?0??0???00???220???????001?????????0-???????????????????????????0010??0001???0??11??23??1?21000-0?0001?11120110?0?1???0?00110101??1????1?0100012000110?001?????100111?1?011?1??010-

Zanabazar_junior ??10000011000001010101001110000001110001????00001000??1???1????????????????111?????00?000100?0-00120100?0000?0???????????????????001100?0????01??0??110001010-0010-??10-???0-?1110???0?001000000-0110-000????????????1100?1010001102101-0010001000001010-0????????????????????????????????????????????????????????????40?1010?00???0??00???2-?0?????????1?12?????????????????????????????????????????????????????????????????????????????????????????????????????????????????????????????????????????????????????1??????0?????????????????????????????????????????????????????20001?????1???????????-?1???????????

Apsaravis_ukhaana ???????0????????????????????????????????????????????????????????????????????????????????????????0?????????????????1?????????????????????????????????????????????????????????????????0??001010011100-0-0??????????????????????????????????????2----??????????????110-1?001?12110??1?11???00??0?????????????????????0???50?1210?0?210?10-0200-??2?0110????????1?1001???3000-000111000001101001100?00011001110001010000111110001????110?11-0?1???????????????????11000-01010--0???1?1?1?000-10????3012--1---1-00000-00000-1-22000??11?11112-2001?????1100???????????11????????11?????110?111?00-?000110?11010?001000-

Archaeopteryx_lithographica 1100001010010001010?1?00?00-000100010001???0000010001100111100021?0-0--1100111110-1101000??0?0-000000-010?00?000???0-210??12?010?0?0??????????0??0?01110001???0???????1001011?00??10010000-?0000-00-0-0?00-0-?1?0?00-0??001010001002001?212010000?00--10-0??1???0?0-??????10??????0?????????0??????????????00?????0??04?????0???20?01??01??1??2?00?0????1?111?00?1?102012-00001100?0001111?110020??1?000010????000?011?10?00?0-?0??01???0?1?22?0?0??2??0000000???00-010???1001?1??0????0-01100?20?1????11???0011210000010?2101001??????11?0110?0??0?00??0??????0010?????00?0002000110?11110011?00?00?0?010?0??010-

Confuciusornis_sanctus 11-000-01?000000----0?01?0--00110011000????00000?0?01?11-111001-1?0-0???1?0????10-??00?0?10??0-000?0????0?00?000??1?????????0??0?????????????????????1?????0?????????????????????0?10??000-?0011000-100?0100-?1?000??0???011---?--1----------2----??------??????0??????????????????????0????0???0??????0??100?????1??05?????0???20?01??1-?0?????010110??????111001110?0????????1???00?11110110020??11001110001?1000011?11?00?0-?01?0????0?1?22?0?0?????000000?1?000-010????0?1?1???????0-??100?300---??1????0010-1?????1?211011?11?????2-20?1?????0??0??1??????00???????01??1??2110???111100??001?10?01??0?0??000-

Rahonavis_ostromi ??????????0???????????0????????????????????????????????????????????????????????????????????????????????????????????????????????????????????????????????????????????????????????????????????????????????????????????????????????????????????????????????????????????????????????????????012011110000000?1001000010010?041?001?000??10120010010020??????1?1112??????????????????1100000110??0????2?????????10001010000??????????????????????????????????00??????01000-21011110011100010000-0111002011??00111--0011210000011121011010001112-20110000100000101111110011100100100012000110100110011011110101110?001010-

Sapeornis_chaoyangensis ??0000000?010??1?1??0???????0??????1?00?????00001000??????11-002?00-???12?0????10-11???0?100?0-0000?0-0????????????????????????????????????????????????????????????????????????????10-?001001010-00-0-0-00-0-?1?101??0??0010100?100?011-2120?2--------?0-0??????0??????????2??????211?????0??????0???????????????????05???01??0?210-0??1-0000??????0??????????????1112010-000?1100?0011011?11012?001?0011100?10000001111100010-10?10111?0?1022?0?020010010000001000-11010-???1?1??010000-0?100?30110101101-01010-10001011010011010?11112-2?11??00100?0????????????1????????11??1001???11100010??0?10??10?0?0??000-

Jeholornis_prima ????????????0??????????????????????1???????????0???0????????????????????1?0???????????????????????????????????1????0??????????????????????????????????????????????????100????????0?????000-?0?????????????????????????????????????1------?--?1?0????---0-0???????????????????????????????2??????????????????0?????????3????1????20?02??02?0?1??????0??????????????11?2??????????0???0?1011?1{01}?0??????????10??001?0??1?111?0?????????????0?1?22?0???????000000????00-110?10??0??1?????????????????????????????????????????????1??1???????????????????????????????????????????????????????1?????????????????????????

Yixianornis_grabaui ???100??2?0?0???????????1??????????????????????0????????????-????????????????????????????????0-?0?????????????????1?????????????????????0????????????????????????????????????????????0?0?0-10????00-????0?????1??0????????10100?101?--1--12??100?0??--10-011????110-???????0????????????02?????00???????????0?????0??07???00????21?????1-?000?2???11???-????1100001003000-00011100?0?110110110???000100??1000100000010111?0?????????????0???22?????????01000??110?0-000?0-?????1???????0-1?1???3012--?????-?1?01210000???20?01?010?????2-201???????0??0???????????11????????1?1???????11??0010?????0???????0???0??

**8. Supplementary References**

1 Perle, A., Norell, M. A., Chiappe, L. M. & Clark, J. M. Flightless bird from the Cretaceous of Mongolia. *Nature* **362**, 623–626, doi:10.1038/362623a0 (1993).

2 Perle, A., Chiappe, L. M., Barsbold, R., Clark, J. M. & Norell, M. Skeletal morphology of *Mononykus olecranus* (Theropoda, Avialae) from the Late Cretaceous of Mongolia. *Am. Mus. Novit.* **3105**, 1–29 (1994).

3 Chiappe, L. M., Norell, M. A. & Clark, J. M. The skull of a relative of the stem-group bird *Mononykus*. *Nature* **392**, 275–278, doi:10.1038/32642 (1998).

4 Chiappe, L. M., Norell, M. A. & Clark, J. M. In *Mesozoic Birds: Above the Heads of Dinosaurs* (eds Chiappe, L. M. & Witmer, L. M.) 87–120 (University of California Press, 2002).

5 Suzuki, S. *et al.* A new specimen of *Shuvuuia deserti* Chiappe et al., 1998 from the Mongolian Late Cretaceous with a discussion of the relationships of alvarezsaurids to other theropod dinosaurs. *Contrib. Sci.* **494**, 1–18 (2002).

6 Karhu, A. A. & Rautian, A. S. A new family of Maniraptora (Dinosauria: Saurischia) from the Late Cretaceous of Mongolia. *Paleontol. J.* **30**, 583–592 (1996).

7 Alifanov, V. R. & Barsbold, R. *Ceratonykus oculatus* gen. et sp. nov., a new dinosaur (?Theropoda, Alvarezsauria) from the Late Cretaceous of Mongolia. *Paleontol. J.* **43**, 94–106, doi:10.1134/s0031030109010109 (2009).

8 Turner, A. H., Nesbitt, S. J. & Norell, M. A. A large alvarezsaurid from the Late Cretaceous of Mongolia. *Am. Mus. Novit.* **3648**, 1–14, doi:10.1206/639.1 (2009).

9 Nesbitt, S. J., Clarke, J. A., Turner, A. H. & Norell, M. A. A small alvarezsaurid from the eastern Gobi Desert offers insight into evolutionary patterns in the Alvarezsauroidea. *J. Vertebr. Paleontol.* **31**, 144–153, doi:10.1080/02724634.2011.540053 (2011).

10 Xu, X. *et al.* A basal parvicursorine (Theropoda: Alvarezsauridae) from the Upper Cretaceous of China. *Zootaxa* **2413**, 1–19 (2010).

11 Xu, X. *et al.* A monodactyl nonavian dinosaur and the complex evolution of the alvarezsauroid hand. *Proc. Natl. Acad. Sci. U.S.A.* **108**, 2338–2342, doi:10.1073/pnas.1011052108 (2011).

12 Xu, X. *et al.* Osteology of the Late Cretaceous alvarezsauroid *Linhenykus monodactylus* from China and comments on alvarezsauroid biogeography. *Acta Palaeontol. Pol.* **58**, 25–46, doi:10.4202/app.2011.0083 (2013).

13 Longrich, N. R. & Currie, P. J. *Albertonykus borealis*, a new alvarezsaur (Dinosauria: Theropoda) from the Early Maastrichtian of Alberta, Canada: implications for the systematics and ecology of the Alvarezsauridae. *Cretac. Res.* **30**, 239–252, doi:https://doi.org/10.1016/j.cretres.2008.07.005 (2009).

14 Novas, F. E. Anatomy of *Patagonykus puertai* (Theropoda, Avialae, Alvarezsauridae), from the Late Cretaceous of Patagonia. *J. Vertebr. Paleontol.* **17**, 137–166, doi:10.1080/02724634.1997.10010959 (1997).

15 Bonaparte, J. F. Los vertebrados fósiles de la Formación Río Colorado, de la ciudad de Neuquén y cercanías, Cretácico superior, Argentina. *Rev. del Museo Argent. de Ciencias Naturales "Bernardino Rivadavia." Paleontología* **4**, 17–123 (1991).

16 Choiniere, J. N. *et al.* A basal alvarezsauroid theropod from the early Late Jurassic of Xinjiang, China. *Science* **327**, 571–574, doi:10.1126/science.1182143 (2010).

17 Averianov, A. & Sues, H.-D. The oldest record of Alvarezsauridae (Dinosauria: Theropoda) in the Northern Hemisphere. *PLoS ONE* **12**, e0186254, doi:10.1371/journal.pone.0186254 (2017).

18 Agnolin, F. L., Powell, J. E., Novas, F. E. & Kundrát, M. New alvarezsaurid (Dinosauria, Theropoda) from uppermost Cretaceous of north-western Patagonia with associated eggs. *Cretac. Res.* **35**, 33–56, doi:https://doi.org/10.1016/j.cretres.2011.11.014 (2012).

19 Carrano, M. T. & Hutchinson, J. R. Pelvic and hindlimb musculature of *Tyrannosaurus rex* (Dinosauria: Theropoda). *J. Morphol.* **253**, 207–228, doi:doi:10.1002/jmor.10018 (2002).

20 Lü, J.-c. *et al.* A new alvarezsaurid dinosaur from the Late Cretaceous Qiupa Formation of Luanchuan, Henan Province, central China. *China Geol.* **1**, 28–35, doi:10.31035/cg2018005 (2018).

21 Martinelli, A. G. & Vera, E. I. *Achillesaurus manazzonei*, a new alvarezsaurid theropod (Dinosauria) from the Late Cretaceous Bajo de la Carpa Formation, Río Negro Province, Argentina. *Zootaxa* **1582**, 1–17 (2007).

22 Naish, D. & Dyke, G. J. *Heptasteornis* was no ornithomimid, troodontid, dromaeosaurid or owl: the first alvarezsaurid (Dinosauria: Theropoda) from Europe. *Neues Jahrb. Geol. Paläontol.* **7**, 385–401 (2004).

23 Xu, X. *et al.* Two Early Cretaceous fossils document transitional stages in alvarezsaurian dinosaur evolution. *Curr. Biol.* **28**, 2853–2860, doi:https://doi.org/10.1016/j.cub.2018.07.057 (2018).
